# BayMeth: improved DNA methylation quantification for affinity capture sequencing data using a flexible Bayesian approach

**DOI:** 10.1186/gb-2014-15-2-r35

**Published:** 2014-02-11

**Authors:** Andrea Riebler, Mirco Menigatti, Jenny Z Song, Aaron L Statham, Clare Stirzaker, Nadiya Mahmud, Charles A Mein, Susan J Clark, Mark D Robinson

**Affiliations:** 1 Institute of Molecular Life Sciences, University of Zurich, Winterthurerstrasse 190, CH-8057 Zurich, Switzerland; 2 Institute of Social and Preventive Medicine, University of Zurich, Hirschengraben 84, CH-8001 Zurich, Switzerland; 3 Department of Mathematical Sciences, Norwegian University of Science and Technology, N-7491 Trondheim, Norway; 4 Institute of Molecular Cancer Research, University of Zurich, Winterthurerstrasse 190, CH-8057 Zurich, Switzerland; 5 Epigenetics Laboratory, Cancer Research Program, Garvan Institute of Medical Research, Sydney 2010, New South Wales, Australia; 6 St Vincent’s Clinical School, University of NSW, Sydney 2052, New South Wales, Australia; 7 Genome Centre, Barts and the London, Queen Mary University of London, Charterhouse Square, London EC1M 6BQ, UK; 8 SIB Swiss Institute of Bioinformatics, University of Zurich, Zurich, Switzerland

## Abstract

Affinity capture of DNA methylation combined with high-throughput sequencing strikes a good balance between the high cost of whole genome bisulfite sequencing and the low coverage of methylation arrays. We present BayMeth, an empirical Bayes approach that uses a fully methylated control sample to transform observed read counts into regional methylation levels. In our model, inefficient capture can readily be distinguished from low methylation levels. BayMeth improves on existing methods, allows explicit modeling of copy number variation, and offers computationally efficient analytical mean and variance estimators. BayMeth is available in the Repitools Bioconductor package.

## Background

DNA methylation (DNAme) is a critical component in the regulation of gene expression, is precisely controlled in development and is known to be aberrantly distributed in many diseases, such as cancer and diabetes [1,2]. In differentiated cells, DNAme occurs primarily in the CpG dinucleotide context. For CpG-island-associated promoters, increases in DNAme (i.e. hypermethylation) induce repression of transcription, while hypomethylated promoters may be transcriptionally active. In cancer, tumor suppressor gene promoters are frequently hypermethylated, and therefore silenced, while hypomethylation can activate oncogenes, which collectively can drive disease progression [3,4]. The detection and profiling of such abnormalities across cell types and patient cohorts is of great medical relevance, both for our basic understanding of how the disease manifests but also for the opportunities of translating this knowledge to the clinic [5]. Epigenetic patterns can be used as diagnostic markers, predictors of response to chemotherapy and for understanding mechanisms of disease progression [6-9]. Acquired epigenetic changes are potentially reversible, which provides important therapeutic opportunities; notably, the US Food and Drug Administration has approved at least four epigenetic drugs and others are in late-stage clinical trials [8].

Four classes of methods are available to profile DNAme genome-wide: chemical conversion, endonuclease digestion, direct sequencing and affinity enrichment. Combinations of techniques are also in use, such as reduced representation bisulfite sequencing (RRBS) [10]. For recent reviews of the available platforms, see [11-13]. Treatment of DNA with sodium bisulfite is the gold standard, giving a single-base readout that preserves methylated cytosines while unmethylated cytosines are converted to uracil [14]. This approach can be coupled with high-throughput sequencing, e.g. whole genome bisulfite sequencing (WGBS), or a ‘genotyping’ microarray (e.g. Illumina Human Methylation 450k array, San Diego, USA [15]). Because WGBS is genome-wide, it inefficiently reveals the methylation status for low CpG density regions [16] and is cost-limiting for larger cohorts; however, recent statistical frameworks allow coverage to be traded for replication [17] and sequencing targeted regions may be a plausible way to increase efficiency [18,19]. Meanwhile, Illumina arrays cover less than 2% of genomic CpG sites and are only available for profiling human DNA, while enzymatic digestion approaches are limited by the location of specific sequences. There is considerable excitement surrounding third-generation sequencing technologies that directly infer methylation status, but these are not yet readily available and generally offer lower throughput [20,21].

An attractive alternative that provides a good tradeoff between cost and coverage, albeit at lower resolution, is affinity capture of methylated DNA in combination with high-throughput sequencing (e.g. methylated DNA immunoprecipitation sequencing (MeDIP-seq) [6,22]). Using affinity capture with antibodies to 5-methylcytosine or methyl-CpG binding domain-based (MBD) proteins, subpopulations of methylated DNA are captured, prepared, sequenced and mapped to a reference genome (see Laird [11]). Åberg *et al*. [23] studied the use of MBD sequencing (MBD-seq) for methylome-wide association studies with 1,500 case–control samples, and proved the potential of MBD-seq as a cost-effective tool in large-scale disease studies. A recent comparative study highlighted that affinity capture methods can uncover a significantly larger fraction of differentially methylated regions than the Illumina 450k array [24]. With appropriate normalization, the density of mapped reads can be transformed to a quantitative readout of the regional methylation level. However, the capability of these procedures to interrogate a given genomic region is largely related to CpG density, which influences the efficiency of capture and can differ from protocol to protocol [16,25,26]. Thus, statistical approaches are needed.

Several methods have been proposed to estimate DNAme from affinity-based DNAme data. For example, MBD-isolated genome sequencing, a variant of MBD-seq, assumes a constant rate of reads genome-wide and uses a single threshold to binarize as methylated or not [27]. State-of-the-art methods, such as Batman [22] and MEDIPS [28], build a linear model relating read density and CpG density, which is then used to normalize the observed read densities. For MeDIP-seq data, the algorithms had similar estimation performance [28], though MEDIPS was considerably more time-efficient. A new tool called BALM uses deep sequencing of MBD-captured populations and a bi-asymmetric-Laplace model to provide CpG-specific methylation estimates [29]. All methods, however, suffer from the same limitations: low capture efficiency cannot easily be distinguished from low methylation level; and, other factors that directly affect read density, such as copy number variation (CNV), are not easily taken into account. For CNV correction, a few possibilities have emerged, such as omitting known regions of amplification [6], adjusting read densities manually [30] and adjusting using the read density from an input sample [29]. Very recently, a method based on combining profiles from MeDIP/MBD-seq and methylation-sensitive restriction enzyme sequencing for the same samples with a computational approach using conditional random fields appears promising [31].

We present a novel empirical Bayes model called BayMeth, based on the Poisson distribution, that explicitly models (affinity capture) read densities of a fully methylated control (e.g. DNA treated with SssI CpG methyltransferase) together with those from a sample of interest. Here, SssI data provide the model an awareness of where in the genome the assay can detect DNAme and the model allows integration of CNV and potentially other estimable factors that affect read density. We have derived an analytic expression for the mean methylation level and also for the variance. Interval estimates, such as credible intervals, can be computed using numerical integration of the analytical posterior marginal distributions. Using MBD-seq for human lung fibroblast (IMR-90) DNA, where ‘true’ methylation levels are available from WGBS, we found favorable performance compared to existing approaches in terms of bias, mean-squared error, Spearman correlation and coverage probabilities. We found that improved performance can even be observed when ignoring SssI data. Model-based SssI correction, however, does not only lead to better performance, but, in addition, data originating from different capture platforms can be compared more easily by propagating the platform-specific uncertainty. Using MBD-seq data for human prostate carcinoma (LNCaP) cells, we showed that directly integrating CNV data provides additional performance gains. The performance with historical data, where no matched SssI sample is available, was demonstrated using data for embryonic stem cells, and colon tumor and normal samples from [32].

## Results and discussion

### BayMeth: A Bayesian framework for translating read densities into methylation levels

DNAme data can be obtained using MBD-seq or a similar affinity enrichment assay. Let *y*_*iS*_ and *y*_*iC*_ denote the observed number of (uniquely) mapped reads for genomic regions *i* = 1, …, *n* for the sample of interest and the SssI control, respectively. Throughout this paper, we use non-overlapping regions (mostly of width 100bp) that have at least 75% mappable bases (see Materials and methods). Let

(1) $$\begin{array}{l} y_{\text{iS}}|\mu_{i},\lambda_{i}∼\text{Poisson}\left(f\times \frac{{\text{cn}}_{i}}{\text{ccn}}\times \mu_{i}\times \lambda_{i}\right),\;\text{and} \end{array}$$

(2) $$\begin{array}{l} y_{\text{iC}}|\lambda_{i}∼\text{Poisson}\left(\lambda_{i}\right), \end{array}$$

with *λ*_*i*_ > 0 and 0 < *μ*_*i*_ < 1. Here, *λ*_*i*_ denotes the region-specific read density at full methylation, *μ*_*i*_ the regional methylation level and *f* > 0 represents the (effective) relative sequencing depth between libraries (i.e. a normalization offset). An approximately linear relation between the copy number state and MBD-seq read density has been established [33]. Hence, if needed, we include a multiplicative offset cn_*i*_/ccn in our model formulation, where cn_*i*_ denotes the copy number state at region *i* and ccn is a cell’s most prominent CNV state (e.g. two in normal cells).

#### Closed-form posterior methylation quantities

In a Bayesian framework, prior distributions are assigned to all parameters. The methylation level (*μ*_*i*_) has support from zero to one. Potential priors include mixtures of beta distributions or a Dirac-Beta-Dirac mixture. In the latter, a beta distribution is combined with point masses placed on zero and on one. The mixture weights can be either unknown or fixed. By default, BayMeth assumes a uniform prior distribution for *μ*_*i*_ (i.e., a beta distribution with both parameters set to 1). For the region-specific density, we assume a gamma distribution, i.e. *λ*_*i*_ ∼ Ga(*α*, *β*) using the shape *α* > 0 and rate *β* > 0 hyperparameters, which are determined in a CpG-dependent manner (see next section). To make inferences for the regional methylation levels, *μ*_*i*_, we integrate out *λ*_*i*_ from the posterior distribution:

$$\begin{array}{ll} p\;\left(\mu_{i}|y_{\text{iS}},y_{\text{iC}}\right) & \;=\;\int_{0}^{\infty }p\left(\lambda_{i},\mu_{i}|y_{\text{iS}},y_{\text{iC}}\right)d\lambda_{i}\\ & \;=\;\int_{0}^{\infty }\;\frac{p\left(y_{\text{iS}}|\lambda_{i},\mu_{i}\right)p\left(y_{\text{iC}}|\lambda_{i}\right)p\left(\lambda_{i}\right)p\left(\mu_{i}\right)}{p\left(y_{\text{iS}},y_{\text{iC}}\right)}d\lambda_{i} \end{array}$$

Notably, p(*y*_*iS*_, *y*_*iC*_) can be calculated analytically [34], so that the marginal posterior distribution:

(3) $$p\left(\mu_{i}|y_{\text{iS}},y_{\text{iC}}\right)=\frac{\mu_{i}^{y_{\text{iS}}}}{W}{\left(1-\frac{E\left(1-\mu_{i}\right)}{\beta +1+E}\right)}^{-\left(\alpha +y_{\text{iS}}+y_{\text{iC}}\right)},$$

is given in closed form with

$$E=f\cdot \frac{{\text{cn}}_{i}}{\text{ccn}}$$

and

$$W=\frac{1}{y_{\text{iS}}+1}\times {\;}_{2}F_{1}\left(y_{\text{iS}}+y_{\text{iC}}+\alpha ,1;y_{\text{iS}}+2;\frac{E}{\beta +1+E}\right)$$

where _2_*F*_1_() is the Gauss hypergeometric function (see page 558 of [35]). The posterior mean and the variance are analytically available (see Additional file 1) and therefore efficient to compute. Credible intervals, which are quantile-based or use the highest posterior density (HPD), can be computed from equation (3). Wald credible intervals are computed on the logit scale, where logit(*μ*_*i*_) = log(*μ*_*i*_ / (1 - *μ*_*i*_)), and then transformed back. These intervals are based on assuming asymptotic normality of the logit methylation estimate. The 95% Wald interval on the logit scale is computed from $\mathrm{logit}\left({\hat{\mu }}_{i}\right)\pm 1.96\cdot {\hat{\sigma }}_{i}$, where ${\hat{\sigma }}_{i}$ is the standard error estimate of $\text{logit}\left({\hat{\mu }}_{i}\right)$. For detailed statistical derivations, also including more general prior distributions for *μ*_*i*_, refer to Additional file 1.

#### Empirical Bayes for prior hyperparameter specification

Our method takes advantage of the relation between CpG density and read depth to formulate a CpG-density-dependent prior distribution for *λ*_*i*_ (and possibly unknown parameters in the prior distribution of *μ*_*i*_). Taking CpG density into account, the prior should stabilize the methylation estimation procedure for low counts and in the presence of sampling variability. All unknown hyperparameters are determined in a CpG-density-dependent manner using empirical Bayes. For each genomic bin of a predetermined size, e.g., 100 bp, we determine the weighted number of CpG dinucleotides within an enlarged window, say 700 bp, around the center of the bin (see Materials and methods and MEDME [36]). Each region is classified based on its CpG density into one of *K*( = 100) non-overlapping CpG density intervals (see *x*-axis tick marks in Additional file 2: Figure S1).

**Figure 1:**
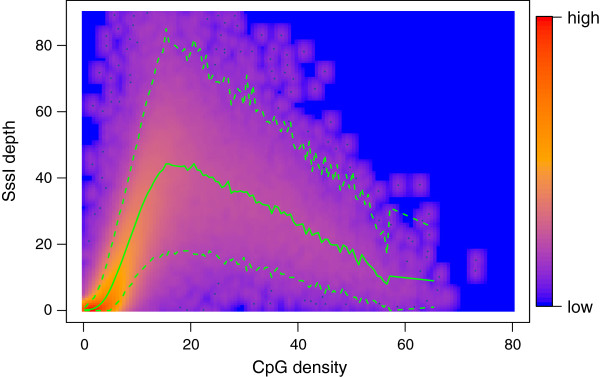
**SssI read depth versus CpG density together with prior predictive distribution.** Smoothed color density representation of SssI read depth versus CpG density together with the mean (green solid line) and 2.5*%* and 97.5*%* quantiles (green dashed lines) of the prior predictive distribution for the SssI control sample. The parameters for this negative binomial distribution were derived using an empirical Bayes approach by maximizing the joint marginal distribution of the IMR-90 and SssI control counts stratified into 100 CpG density groups. Only counts from bins with a mappability larger than 0.75 were considered.

For each class separately, we derive the values for the hyperparameters under an empirical Bayes framework using maximum likelihood. Both read depths, from the SssI control and the sample of interest, are thereby taken into account, since *λ*_*i*_ is a joint parameter affecting both. We end up with *K* parameter sets. To illustrate the (known) relation between SssI read count and CpG density, we considered only the SssI Poisson model (equation (2)) and derived the prior predictive distribution by integrating *λ*_*i*_ out. This results in a negative binomial distribution for each CpG class (see Figure 1, which uses SssI data from [37] that are later used in the analysis of the IMR-90 cell line).

#### SssI-free BayMeth

Although we recommend collecting at least a single SssI sample under the same protocol as the data of interest, BayMeth can, in principle, be run without a SssI-control sample. The statistical framework then only involves the Poisson model for the sample of interest (equation (1)) and no longer borrows strength from the information included in the SssI-control sample (equation (2)). The same model is used in the analysis of under-reported count data in economics [34,38,39], where it is assumed that the number of registered purchase events under-reports the actual purchase rate. According to Fader and Hardie [34], the parameters *λ*_*i*_ and *μ*_*i*_ are identifiable assuming that the gamma and beta prior distributions are able to capture unobserved heterogeneity in the read density rate and the methylation level. As in the framework with SssI data, parameters for the gamma prior distributions of the region-specific read density *λ*_*i*_ can still be determined in a CpG-density-dependent manner using empirical Bayes; however, no information can be borrowed from the fully methylated control. Furthermore, the determination of the normalizing offset *f* is more involved. Interpretation moves from the (effective) relative sequencing depth between libraries to the number of bins potentially ‘at risk’ of being methylated in the sample of interest. Here, we fix *f* at the 99% quantile of the number of reads. The results for the posterior mean and variance of the methylation level change accordingly (see Additional file 1).

### Analysis of affinity capture methylation data with a matched SssI sample

For the following, we used BayMeth to affinity capture methylation data. We collected a SssI-control sample under the same conditions (e.g. same elution protocol) used for the samples of interest. Hence, both data components are matched.

#### BayMeth improves estimation and provides realistic variability estimates

To take advantage of the single-base-resolution high-coverage methylome obtained using WGBS by Lister *et al*. [40], we generated IMR-90 MBD-seq data under the same protocol as our previously published SssI MBD-seq dataset [37], i.e. using a single fraction with a high salt elution buffer (MethylMiner™). We applied BayMeth to chromosome 7, which consists of 1,588,214 non-overlapping bins of width 100 bp. Only bins with at least 75% mappable bases were included, so we analyzed 1,221,753 bins (approximately 77%).

We ran BayMeth in two configurations: (1) incorporating SssI information and assuming a uniform prior between zero and one for the methylation parameter and (2) ignoring SssI information and assuming a Dirac-Beta-Dirac mixture prior distribution for the methylation parameter. That means we set a point mass on zero and on one, giving each a prior weight of 10%. The parameters of the central beta component were assumed to be unknown. The normalizing offset *f* = 0.581 for configuration 1 was found by calculating a scaling factor between highly methylated regions in IMR-90 relative to the SssI control (see Materials and methods and Additional file 2: Figure S2). The prior parameters for the gamma distributions and the parameters of the beta distribution in configuration 2 were determined by empirical Bayes, as discussed above (see also further details in Materials and methods).

We compared the results from BayMeth, both ignoring and taking advantage of the SssI control, to those obtained from Batman [22], MEDIPS [28] and BALM [29]. To provide plausible uncertainty estimates with Batman, we increased the default number of generated samples from 100 to 500. The WGBS data, here considered to be the ‘truth’ (at suitable depth), and the CpG-specific BALM methylation estimates were collapsed into 100-bp bin estimates (see Materials and methods) to match the estimates from MEDIPS, Batman and our approach. For about 53% (645,451) of the analyzed bins, no WGBS data were available (largely due to the lack of CpG sites). For 17,259 bins, no methylation estimates were provided by Batman, so that in total, algorithm comparisons were conducted on the remaining 559,043 bins.

**Figure 2:**
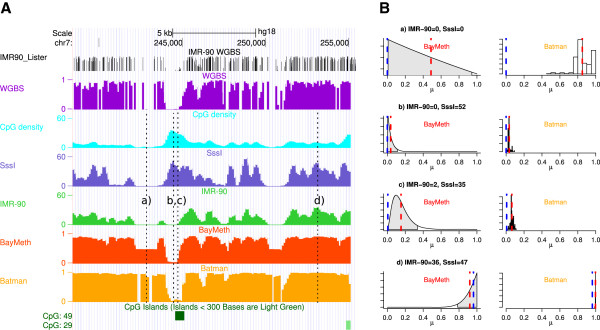
**Example data tracks for IMR-90 chromosome 7. (A)** WGBS methylome (black) per CpG-site and per 100-bp bin (purple) as obtained by Lister and others [40]. CpG density (light blue), and read counts for SssI-treated DNA (blue) and IMR-90 cells (green) obtained by MBD-seq based on 100-bp non-overlapping bins are shown. Methylation estimates for BayMeth (red) and Batman (orange) are provided. **(B)** Detailed posterior information for BayMeth and Batman for four specific bins of panel **A** (denoted a, b, c and d). For BayMeth, the posterior marginals together with 95% HPD credible intervals (shaded gray) are shown. The posterior samples obtained by Batman are plotted as histograms. For both approaches the posterior mean is indicated (red dashed line) together with the true WGBS-derived methylation estimate (blue dashed line). chr, chromosome; kb, kilobase; WGBS, whole genome bisulfite sequencing.

The behavior of BayMeth (including SssI-information) and Batman is illustrated using an example region of chromosome 7 (see Figure 2A). WGBS levels, CpG density and read counts per 100-bp region of MBD-seq SssI and IMR-90 samples are shown. As expected, the number of reads in the SssI control is related to the CpG density, whereas the read density in the IMR-90 MBD-seq data is modulated by both the region-specific density and the DNAme level. Regions lacking both IMR-90 and SssI reads suggest inefficient MBD-based affinity capture (e.g. region a). Figure 2B shows posterior samples from Batman and inferred posterior distributions from BayMeth. For region a, Batman’s posterior samples are concentrated between 0.7 and 1 (mean equal to 0.85). In contrast, BayMeth returns a mean methylation level of 0.49 together with a large 95% HPD interval (0,0.94), reflecting the uncertainty from having no SssI reads sampled. The credible interval covers nearly the entire interval, reflecting that no reliable estimate can be made for this bin due to inefficient capture. For regions with no IMR-90 reads but efficient capture (e.g. region b), both BayMeth and Batman provide sensible posterior marginal distributions and low DNAme estimates. If there are a small number of reads for IMR-90 with efficient capture (e.g. region c), the BayMeth posterior marginal is more disperse than Batman’s, while both are close to zero. Region d has a high number of reads for both samples and a true methylation level around 0.95. This level is covered by the 95% HPD region of BayMeth, while it lies outside the density mass obtained by Batman, which overestimates this region.

Table 1 summarizes the estimation performance for chromosome 7 using mean bias (difference between the posterior mean $\hat{\mu_{i}}$ and the true value *μ*_*i*_), mean of squared differences (MSE) and Spearman correlation for BayMeth, BayMeth ignoring SssI-information, Batman, MEDIPS and BALM. To account for uncertainty in the WGBS estimates, we applied a threshold on the depth. We assessed the performance using bins with at least 33 WGBS reads (unmethylated and methylated) corresponding to the 25% quantile of depth in the truth, which gave 414,352 bins. The results were stratified into five groups according to depth in the SssI control, which should represent a surrogate of the capture efficiency. The first group [ 0,4] encompasses primarily low-CpG regions that are not well captured in MBD experiments, while the high (27,168] group represents primarily CpG island regions. On average, Batman tended to overestimate DNAme while MEDIPS and BALM tended to underestimate it. BayMeth, in contrast, was almost unbiased. The smaller bias in the point estimates obtained by BayMeth was also reflected in the MSE. For all methods, the MSE decreased with higher SssI depth, as expected due to the efficiency of capture. For all depth groups, BayMeth had the highest correlation with the WGBS estimates, which increased with higher SssI depth. The SssI-free version of BayMeth performed comparable to the other approaches, with slightly smaller bias and MSE; however, there was a smaller correlation for bins with low SssI depth.

**Table 1:** Performance assessment for IMR-90 analysis (chromosome 7)

**SssI depth**	**Number of bins**	**Method**	**Mean bias**	**Mean of squared differences**	**Spearman correlation**	**Wald**	**Highest posterior density**	**Quantile**
[ 0,4]	305,638	BayMeth	-0.04	0.08	0.36	0.74	0.89	0.89
		BayMeth (SssI-free)	-0.19	0.20	0.23	—	—	0.24
		Batman	0.22	0.14	0.31	—	—	0.43
		MEDIPS	-0.38	0.26	0.29	—	—	—
		BALM	-0.48	0.33	0.32	—	—	—
(4,7]	22,196	BayMeth	0.05	0.05	0.65	0.84	0.88	0.87
		BayMeth (SssI-free)	-0.01	0.08	0.42	—	—	0.68
		Batman	0.16	0.07	0.61	—	—	0.34
		MEDIPS	-0.23	0.11	0.45	—	—	—
		BALM	-0.27	0.15	0.60	—	—	—
(7,14]	28,871	BayMeth	0.06	0.04	0.69	0.84	0.86	0.86
		BayMeth (SssI-free)	0.02	0.05	0.57	—	—	0.79
		Batman	0.16	0.07	0.65	—	—	0.28
		MEDIPS	-0.21	0.10	0.49	—	—	—
		BALM	-0.21	0.11	0.66	—	—	—
(14,27]	28,928	BayMeth	0.05	0.03	0.76	0.81	0.85	0.82
		BayMeth (SssI-free)	0.08	0.04	0.72	—	—	0.70
		Batman	0.15	0.06	0.73	—	—	0.23
		MEDIPS	-0.20	0.09	0.59	—	—	—
		BALM	-0.15	0.07	0.75	—	—	—
(27,168]	28,719	BayMeth	0.02	0.03	0.79	0.73	0.86	0.78
		BayMeth (SssI-free)	0.11	0.04	0.77	—	—	0.48
		Batman	0.11	0.05	0.75	—	—	0.20
		MEDIPS	-0.22	0.10	0.67	—	—	—
		BALM	-0.14	0.06	0.76	—	—	—

Results are shown for bins with a truth depth larger than the 25% quantile (cutoff is 33 reads), stratified into five groups by SssI depth. Shown are the number of bins per group, mean bias, MSE, Spearman correlation and coverage probabilities at 95% level.

**Figure 3:**
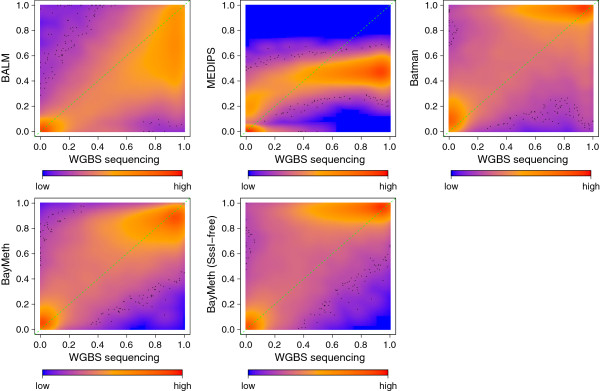
**Regional methylation estimates for IMR-90 chromosome 7.** Smoothed color density representation of regional DNAme estimates for BALM, MEDIPS, Batman, BayMeth and BayMeth ignoring SssI information, plotted against WGBS methylation levels for the 75% of bins with the largest depth in the truth (cutoff was 33 reads) where the depth in the SssI control was (27,168]. In addition the *y* = *x* line (green dashed line) is shown. Black points indicate outliers. WGBS, whole genome bisulfite sequencing.

A smoothed density representation of regional methylation estimates for the highest SssI depth group, namely (27,168], plotted for all methods against the true WGBS methylation levels is shown in Figure 3. Overall, BayMeth provides the most accurate point estimates. The overestimation by Batman and underestimation by MEDIPS and BALM are obvious, while the BayMeth errors vary almost symmetrically. Comparing BALM CpG-wise to WGBS leads to similar conclusions as for the bin-specific setting (results not shown). The pattern for the SssI-free BayMeth estimation (i.e. overestimation) is similar to Batman, which may be expected given that no information was drawn from the SssI sample.

**Figure 4:**
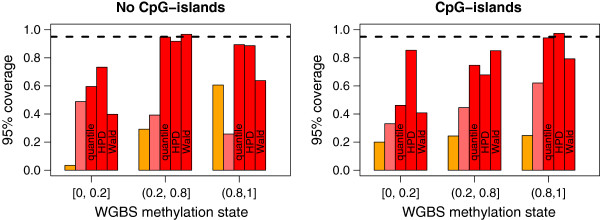
**Coverage probabilities stratified by CpG island status and true methylation level.** Coverage probabilities (frequency in which the true value is within a predefined credible interval) at the 95% level are shown for the 75% of bins with the largest depth in the truth (cutoff was 33 reads) for Batman (orange), BayMeth ignoring SssI control information (light red) and BayMeth (red). Three different types of credible intervals (quantile-based, Wald and HPD) are shown for BayMeth, while for Batman and the SssI-free version of BayMeth only quantile-based intervals are available. MEDIPS and BALM do not return any uncertainty estimates. The nominal coverage value is indicated (black dashed line) as a reference. Genomic regions were stratified by CpG density using the threshold of 12.46, which separates CpG islands from non-CpG islands; compare Additional file 2: Figure S1. Further stratification by the true methylation level as derived from WGBS [40] is provided. HPD, highest posterior density; WGBS, whole genome bisulfite sequencing.

To assess the calibration, we computed coverage probabilities (the frequency that the true methylation value is captured within a credible interval). Stratified by the true WGBS methylation level, Figure 4 shows coverage probabilities at the 95% level for regions deemed to be inside or outside a CpG island (Additional file 2: Figure S1). HPD intervals and quantile-based and Wald-based credible intervals were computed for BayMeth while only quantile-based credible intervals were available for Batman. Coverage probabilities cannot be obtained from the output from MEDIPS and BALM. As mentioned, Batman tended to underestimate the variance, resulting in lower coverage probabilities for the WGBS values. In contrast, BayMeth’s coverage probabilities were much closer to the nominal levels and seemed to be stable across the stratification. For SssI-free BayMeth, quantile-based credible intervals were computed and these were generally better than those provided by Batman (see Table 1 and Figure 4), indicating a more realistic methylation estimation.

For the same stratification, Additional file 2: Table S1 shows the mean bias for BayMeth, Batman, MEDIPS and BALM. While the latter two had a low mean bias for bins where the truth was within [ 0,0.2], Batman performed best for highly methylated bins. BayMeth performed well for bins where the true methylation level was intermediate or high. Like Batman, reasonable estimates were obtained over the whole range of methylation states when considering bins in CpG islands. When interpreting the mean bias, the uncertainty around the obtained estimates should be taken into account and hence the results should be set into context with Figure 4. Combining bias and calibration, BayMeth performed well and seems to be better than the existing approaches.

#### Copy number variation-aware BayMeth improves estimation of DNA methylation for prostate cancer cells

**Figure 5:**
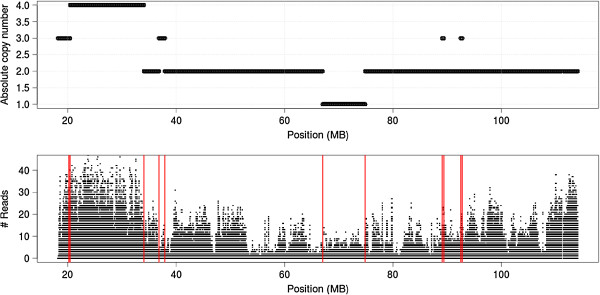
**Relation between copy number state and regional affinity enrichment.** Top: Copy number estimates for the LNCaP cell line obtained by the PICNIC [55] algorithm for 100-bp bins across human chromosome 13 with a mappability of at least 75%. Bottom: Read counts of affinity capture sequencing data for the same bins. MB, megabase.

In the following, we illustrate the benefits of directly integrating CNV information into a cancer MBD-seq dataset. We applied our methodology to the autosomes of the LNCaP cell line. To illustrate the reason for this adjustment, Figure 5 shows the estimated copy number across chromosome 13 (with many non-neutral regions), together with tiled MBD-seq read counts. The copy number estimates were derived using the PICNIC algorithm for Affymetrix genotyping arrays (see Materials and methods). Although read densities at a specific genomic region (again, 100-bp non-overlapping bins) were influenced by a combination of effects (e.g. DNAme and CpG density), a relation between CNV and the number of reads is clearly visible. In particular, a difference in read counts between regions with four copies and those with smaller copy numbers is apparent. We adjusted for this bias through a multiplicative offset cn_*i*_/ccn, where the prominent state was four copies, i.e., ccn = 4 in equation (1) (see Additional file 2: Figure S3). This also assumes that the SssI sample was for a ‘normal’ copy genome. In addition, regions from this state (cn_*i*_ = 4) were used to determine the normalizing offset *f* (here, estimated to be 0.712).

The read depth stratified by copy number state together with mean and median estimates is shown in Additional file 2: Figure S4. In particular, for the three most frequent CNV states (2 to 4), the read densities scale approximately linearly with CNV (with a slope of 1), which justifies the structure of our multiplicative offset. Copy number offsets are given in Table 2.

**Table 2:** Copy number specific offset

	**1**	**2**	**3**	**4**	**5**	**6**	**7**	**8**
Combined offset	0.178	0.356	0.534	0.712	0.889	1.067	1.245	1.423

Copy number specific offsets defined as *f* × cn_*i*_/ccn derived for 100-bp non-overlapping bins of LNCaP autosomes, which have a mappability of at least 75%. Note that *f* is only derived based on bins with the most common copy number state four.

**Figure 6:**
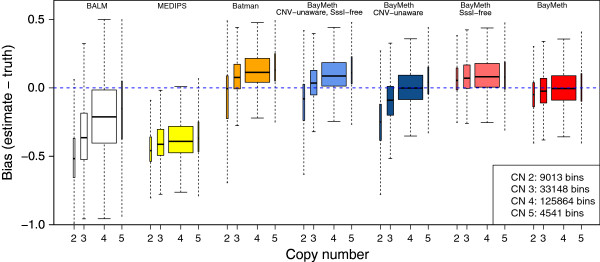
**Bias of LNCaP methylation estimates compared to 450k array beta values.** Box plots for bias (estimated methylation level minus 450K array beta value) for BALM (white), MEDIPS (yellow), Batman (orange), CNV-unaware and SssI-free BayMeth (light blue), CNV-unaware BayMeth (dark blue), SssI-free but CNV-aware BayMeth (light red) and CNV-aware BayMeth (red) stratified by copy numbers 2 to 5. (Outliers are not shown.) The width of the boxes is proportional to the percentage of bins (the legend gives the absolute numbers) for the copy number class. A uniform prior for the methylation level was used taking SssI information into account. In the SssI-free version a Dirac-Beta-Dirac mixture with weights fixed to 0.1, 0.8 and 0.1 was used. The results are shown genome-wide for 100-bp bins with at least 75% mappability and where the true methylation estimate is larger than 0.5. A threshold of 13 was applied for the depth of SssI. The blue dashed line indicates a bias of zero. CN, copy number; CNV, copy number variation.

The box plots in Figure 6 illustrate the bias of DNAme point estimates by integer CNV state (2 to 5) for the different methods. Here, we used the Illumina Human Methylation 450k array as the true methylation (see Materials and methods), since methylation status should be unaffected by CNV [41]. To emphasize that copy number class 4 is the most prominent state, we set the width of the boxes proportional to the percentage of bins that belong to the corresponding copy number classes. Because CNV only affects MBD capture for methylated regions, we restricted this comparison to bins where the true methylation state is larger than 0.5 and we applied a threshold of 13 (the median after excluding bins with a low depth of [ 0,4]) to the number of reads in the SssI-control to select for regions where MBD-seq has good performance. As for the IMR-90 data, MEDIPS and BALM tended to underestimate while Batman tended to overestimate. For BayMeth we show four different approaches as combinations of neglecting CNV or SssI information. As previously, we used a uniform prior for the methylation level when taking advantage of the SssI sample, and a Dirac-Beta-Dirac mixture with fixed weights (0.1, 0.8, 0.1) but unknown beta parameters in the SssI-free case. In the SssI-free version the normalization offset *f* was determined as the 99% percentile of the number or reads for the sample of interest having copy number state 4, while the reads of all bins were used when neglecting the CNV information. Without the additional multiplicative offset (i.e. without cn_*i*_ ≡ ccn) to account for CNV, BayMeth produced biased estimates, predictably by CNV state. After including the copy-number-specific offset, these copy number specific biases almost disappeared, though the SssI-free version still produced a slight overestimation.

**Figure 7:**
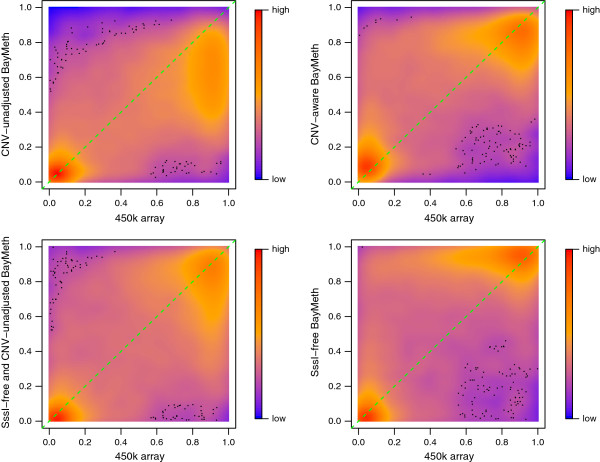
**Effect of adjusting for CNV for the LNCaP cell line.** Smoothed color density representation of methylation estimates for copy number state 2 derived by BayMeth compared to 450k array beta values. A threshold of 13 was applied for the depth of SssI, which gives 61,969 bins, of which we have for 18,010 100-bp bins a beta value and BayMeth estimate. In addition the *y* = *x* line (green dashed line) is shown. Black points indicate outliers. Top left: CNV-unaware BayMeth; top right: CNV-aware BayMeth; bottom left: SssI-free and CNV-unaware BayMeth; bottom right: SssI-free BayMeth. CNV, copy number variation.

A smoothed scatterplot illustrating the benefits of including the copy-number-specific offset is shown in Figure 7 for copy number state 2. In particular, bins that have been falsely underestimated (due to having two copies instead of four) have been corrected (see the top-right panel). Due to overestimation in the SssI-free version (bottom left), the methylation estimates for copy number state 2 do not show such a strong bias. Adjusting for CNV in this case slightly increased the bias (bottom right). Table 3 shows the mean bias, MSE and Spearman correlation for the different approaches stratified by copy number state. In all measures, the CNV-aware standard version of BayMeth (including SssI) performed best. While the differences in the correlation estimates were small, clear advantages can be seen in terms of bias and MSE when compared to Batman, MEDIPS or BALM. In contrast to the other approaches, the bias and MSE performance estimates are almost constant over the different copy number states and are close to zero.

**Table 3:** Performance assessment for LNCaP analysis by copy number

**Copy number**	**Number of bins**	**Method**	**Mean bias**	**Mean of squared differences**	**Spearman correlation**
2	18,010	BayMeth	0.04	0.04	0.78
		BayMeth (SssI-free)	0.08	0.05	0.79
		BayMeth (CNV-unaware)	-0.11	0.06	0.78
		BayMeth (SssI-free, CNV-unaware)	-0.05	0.05	0.79
		Batman	0.03	0.06	0.74
		MEDIPS	-0.23	0.11	0.76
		BALM	-0.29	0.16	0.78
3	65,982	BayMeth	0.05	0.04	0.80
		BayMeth (SssI-free)	0.09	0.05	0.80
		BayMeth (CNV-unaware)	-0.01	0.04	0.80
		BayMeth (SssI-free, CNV-unaware)	0.05	0.04	0.80
		Batman	0.11	0.06	0.77
		MEDIPS	-0.19	0.09	0.76
		BALM	-0.20	0.10	0.79
4	256,074	BayMeth	0.05	0.04	0.81
		BayMeth (SssI-free)	0.10	0.05	0.81
		BayMeth (CNV-unaware)	0.05	0.04	0.81
		BayMeth (SssI-free, CNV-unaware)	0.11	0.06	0.81
		Batman	0.16	0.08	0.79
		MEDIPS	-0.17	0.09	0.76
		BALM	-0.12	0.07	0.80
5	11,790	BayMeth	0.04	0.03	0.83
		BayMeth (SssI-free)	0.07	0.05	0.82
		BayMeth (CNV-unaware)	0.09	0.04	0.83
		BayMeth (SssI-free, CNV-unaware)	0.12	0.06	0.82
		Batman	0.18	0.08	0.80
		MEDIPS	-0.12	0.07	0.80
		BALM	-0.08	0.05	0.82

Results are shown for 100-bp bins with a mappability of at least 75% stratified into the four most frequent copy number states. A threshold of 13 was applied for the depth of the SssI-control. Taking SssI information into account a uniform prior for the methylation level was used. In the SssI-free version a Dirac-Beta-Dirac mixture with weights fixed to 0.1, 0.8 and 0.1 was used. CNV, copy number variation; MSE, mean of squared differences.

#### Improved correlation across methylation kits on IMR-90 DNA

**Figure 8:**
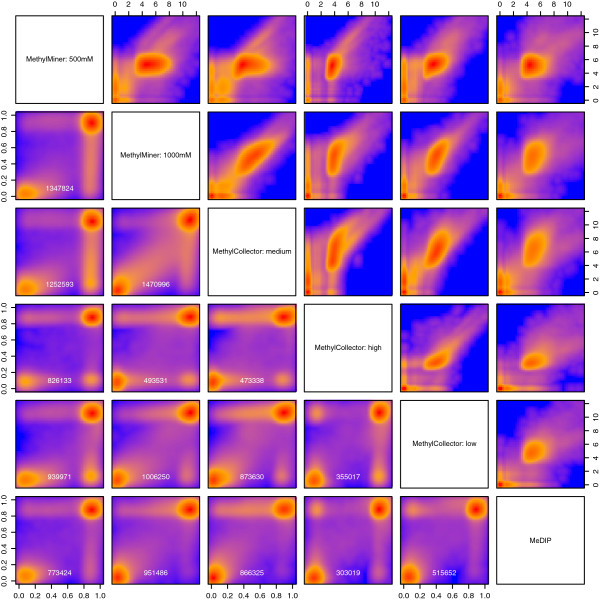
**Comparison of raw IMR-90 data and methylation estimates obtained by different methylation kits.** Genomic bins (100 bp) with a mappability larger than 75% for which the predicted HPD credible interval width was smaller than 0.4 were selected. For these bins the upper triangle of panels shows the smoothed color density (from blue for low density to red for high density) of the raw counts and the lower triangle of panels shows the estimated methylation levels obtained by different methylation kits against each other. The number of bins is given in white in the panels in the lower triangle.

One potential advantage of the proposed model-based SssI correction is that data originating from different capture platforms can be more easily compared. In this situation, propagation of the uncertainty becomes important, since methods to capture methylated DNA have different CpG-dependent affinities and therefore different estimation precisions. To demonstrate this, we captured methylated DNA from IMR-90 and SssI DNA using six approaches: low, medium and high salt elutions from MethylCap Kit™, 500 nM and 1,000 nM salt fractions from MethylMiner™ and MeDIP. Autosomes were analyzed with BayMeth using specific SssI data for each kit. The derived M versus A plots (M: log-fold change; A: log-read-depth) together with the normalizing offsets *f* obtained for each sample are shown in Additional file 2: Figure S5. Unusual high counts were excluded in the derivation of the prior parameters [42], but methylation estimates were derived for all bins. For bins where the estimated credible interval width (HPD) was smaller than 0.4, Figure 8 compares the unnormalized read densities for the six kits (upper triangle of panels) and the obtained methylation estimates (lower triangle of panels). Clearly, capture affinities across the six kits vary drastically, and the SssI-based correction makes the comparison much clearer. In addition, the SssI data from this collection of platforms could be combined by other researchers with their in-house data, assuming similar procedures have been followed (see Discussion), allowing them to benefit from the SssI-based read density correction from BayMeth.

### Analysis of affinity capture methylation data without a matched SssI sample

Next, we applied (default) BayMeth to the MethylCap sequencing data of [32], provided at [43], and referred to as the ‘Bock’ data. Absolute read densities were available for (non-overlapping) 50-bp bins for four samples: HUES6 ES cell line, HUES8 ES cell line, colon tumor tissue and colon normal tissue (same donor as for the colon tumor tissue). There were no matched SssI samples available for these data. To take advantage of BayMeth in analyzing these data, we used a non-matching SssI sample, but one chosen to be maximally compatible to the preparation conditions of the Bock data [32] (i.e. MethylCap with a low salt concentration of 200 mM NaCl). Regions from the data available were converted to hg19 coordinates using liftOver (see Additional file 3 for details). Although there were still slight differences in the preparation of the samples of interest and the SssI sample, which arise from the different read lengths (36 bp versus 75 bp, respectively) and read extensions (300 bp versus 150 bp, respectively) used before the read frequencies were calculated, we regard the SssI sample as a reasonably suitable control for running BayMeth. We analyzed all autosomes after removing bins that have no read depth in any of the four samples, leading to 42,955,764 bins. As in the previous analyses, we restricted our attention to bins that have at least 75% mappable bases, of which there were 37,013,409 or 86% of all bins. A detailed description of all data preparation steps and the data analysis using BayMeth based on the R package Repitools is given in Additional file 3. We compared the methylation estimates obtained using BayMeth with RRBS data available from the Bock study [32]. As in the methylation kit analysis, we masked unusual high counts in the derivation of the prior parameters as they sometimes cause problems in the numerical optimization routine; however, methylation estimates were derived for more than 99.5*%* of these masked bins. Interestingly, several high count regions could be explained by unannotated high copy number regions; see Pickrell *et al*. [42].

**Figure 9:**
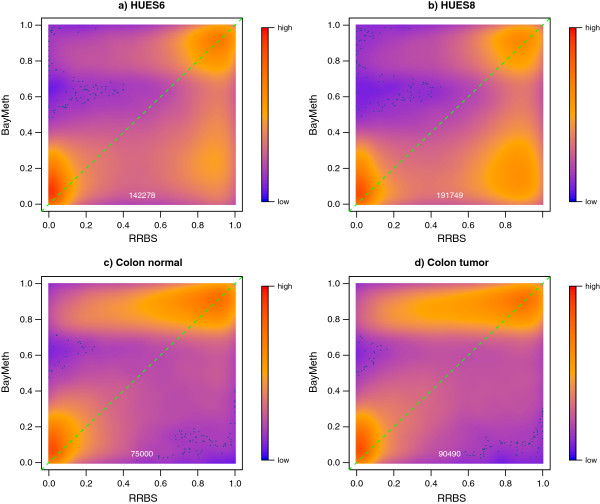
**Regional methylation estimates for samples of Bock data.** Smoothed color density representation of regional DNAme estimates of BayMeth, plotted against RRBS methylation levels, where the estimated standard deviation of BayMeth is smaller than 0.15 for bins with more than 20 reads for RRBS and at least a depth of 10 in the SssI control. **(a)**-**(d)** Single samples from the original study. The number of bins for each sample is shown at the bottom center of the panels. RRBS, reduced representation bisulfite sequencing.

**Figure 10:**
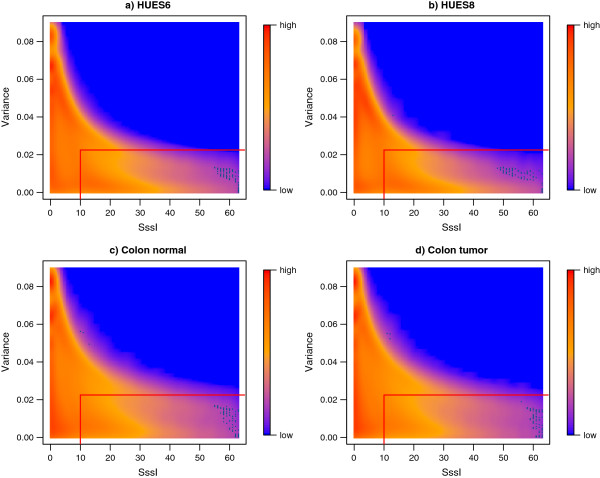
**Regional variance estimates versus SssI control for Bock data.** Smoothed color density representation of variance estimates obtained by BayMeth versus number of reads in the SssI control for a read depth larger than 20 in RRBS. The red boxes contain the bins used in Figure 9, which have a depth of at least 10 in SssI and a standard deviation smaller than 0.15, i.e. a variance smaller than 0.025. **(a)**-**(d)** Single samples from the original study.

Methylation estimates were obtained for about 37 million bins each of width 50 bp, though RRBS estimates were only available for approximately 4% of these bins. We assessed the performance of BayMeth using bins where the depth in the RRBS was larger than 20. Furthermore, we focused on bins where we believe in the SssI control, that means where the read depth is at least 10. Figure 9 shows regional methylation estimates obtained by BayMeth compared to RRBS-derived methylation levels for all four samples of interest where the corresponding posterior standard deviation was smaller than 0.15. In particular, low methylation levels were predicted well for all samples. While high methylation levels were partly underestimated by BayMeth for the human embryonic stem cell line HUES8, estimates for HUES6, colon tumor and color normal tissue reproduce the true methylation for all levels. Although, in the latter two, slight overestimation is visible. This was partly caused by bins for which there was a low read depth in SssI but extreme depth in the sample of interest. BayMeth predicted these bins comprehensibly with high precision (low standard deviation), which may, however, not coincide with the RRBS estimates. Figure 10 shows regional posterior variances obtained by BayMeth compared to SssI depth for bins where the depth in the RRBS was larger than 20. The posterior variance decreased with increasing SssI depth. However, the range of posterior variances for low SssI depth is large. The red boxes contain the bins illustrated in Figure 9. Of note, comparisons to other methods were not possible for the Bock data, since we did not have access to the raw reads.

### Discussion

DNAme plays a crucial role in various biological processes and is known to be aberrant in several human diseases, such as cancer. There are now a multitude of methylation profiling platforms, each with inherent advantages and disadvantages. Bisulfite-based approaches are considered the gold standard since they allow quantification at single-base resolution. However, applied genome-wide, this technique can be inefficient and expensive, in terms of CpGs covered per read or base sequenced [5,16]. On the other hand, approaches based on affinity capture, such as MBD and MeDIP, combined with sequencing seem to provide a good compromise between cost and coverage, albeit at lower resolution. Thus, we consider MBD-seq and its variants to be an attractive alternative and have developed an efficient data analytic approach to facilitate their use. In addition, MBD-seq has recently been used with only hundreds of nanograms of starting DNA, thus making it applicable to a wider range of studies, such as clinical samples [44].

The key to our proposed method is the use of methylated DNA captured from a fully methylated SssI control. To facilitate accurate transformation of read counts into methylation, we recommend this sample is collected under the same conditions (e.g. same elution) used for the samples of interest. In our analyses, we used commercially available SssI-treated DNA [26,37] for the MBD-seq experiments and used the 450k platform to verify that the overwhelming majority of CpG sites were indeed methylated (see Additional file 2: Figure S6). Similarly, such a sample can be constructed directly and inexpensively [45].

Our proposed method, BayMeth, is a flexible empirical Bayes approach, which transforms read densities into regional methylation estimates. Our model is based on a Poisson distribution and takes advantage of SssI control data in two ways: (i) we model SssI data jointly with data from a sample of interest to preserve the linearity of the methylation estimation and (ii) we explicitly get information about the region-specific read density as a function of CpG density. Our method is similar in principle to MEDME, which was applied to fully methylated MeDIP microarray intensities [36]. However, our approach necessarily modifies the assumptions for count data (i.e. read densities versus probe intensities) and is effectively a compromise between the global fit that MEDME implements and a region-specific correction. We showed that BayMeth performed better than state-of-the-art techniques for MBD-seq data, using multiple datasets where independent true methylation levels were available from WGBS or bisulfite-based methylation arrays. In general, MEDIPS and BALM underestimate the methylation levels and do not offer variability estimates. Batman performs reasonably well, but our results suggest that its variability estimates are generally underestimated and the method is very computationally demanding. Our model performed best in point estimation and was the only method to provide reasonable interval estimates. BayMeth uses analytic expressions for the posterior marginal distribution and the posterior mean and variance, avoiding computationally expensive sampling algorithms. Furthermore, we can explicitly integrate existing CNV data, which gives an improvement when applied to cancer datasets. CNV adjustments may be possible with existing approaches such as Batman or MEDIPS, based on ad hoc transformations of the read counts (e.g. see [30]), but are not included within the model formulation. In contrast, our model preserves the count nature of the data. To adjust the modeled mean for effects arising due to library composition or CNV, we introduced a normalization offset. This strategy is quite general and could be extended beyond composition and CNV (e.g. see [33,46]).

A conceptually similar Bayesian hierarchical model, which uses MCMC sampling, has been proposed for methyl-seq experiments where methylation levels are derived based on enzymatic digestion using two enzymes [47]. A separate Poisson model is assumed for the tag counts of each enzyme. The models are linked through a shared parameter. One Poisson model contains a methylation level parameter *μ*, assumed to be uniformly distributed *a priori*. Our model may find application in this domain. In the applications presented here, a uniform prior distribution for the methylation level was observed to perform best when taking SssI information into account, while a mixed prior of a point mass at zero and at one, combined with a beta distribution, performed best when ignoring SssI information. The analytical expressions for the mean, variance and posterior marginal distribution are also available when using a mixture of beta distributions (see Materials and methods). Therefore, context-specific information, such as CpG density or the position relative to transcriptional features, could be incorporated into the prior distribution for the methylation level. We have tried various weighted mixtures of two or three beta distributions that build in contextual information; however, these did not outperform the uniform prior when borrowing strength from the SssI sample. The reason probably lies in the fact that there is only one data point for each methylation parameter. Hence, when using an informative prior distribution for the methylation level, it is very difficult for the data to overcome this prior guess.

It is well known that methylation levels are dependent within neighboring regions. Thus, a potential improvement may involve modeling correlation between neighboring genomic bins. One approach might be Gaussian Markov random fields [48]; however, the analytical summaries are lost, so the gain in performance may not justify the more complex model and associated computational cost.

BayMeth may also be regarded as a preprocessing step in differential methylation analysis. The uncertainty in methylation estimates obtained by BayMeth could be propagated to a downstream analysis, which may lead to improved inferences on differential methylation.

## Conclusions

BayMeth is an empirical Bayes approach that uses a fully methylated (SssI treated) control sample to transform observed read counts into regional methylation levels. BayMeth can be applied to methylated DNA affinity enrichment assays (e.g MBD-seq, MeDIP-seq) and improves on existing methods. Inefficient capture can readily be distinguished from low methylation levels by means of larger posterior variances. Furthermore, copy number variation data can be explicitly integrated, which offers improvement when applied to cancer datasets. Notably, BayMeth offers computationally-efficient analytic expressions for the mean and variance of the methylation level. A software implementation is freely available in the Bioconductor Repitools package.

## Materials and methods

### Methyl binding domain sequencing data for IMR-90, LNCaP and SssI DNA

We used LNCaP and SssI MBD-seq data and Affymetrix genotyping array data (LNCaP only) from Robinson *et al*. [37]. The data can be found at [49] under accession number [GEO:GSE24546]. Similarly, IMR-90 MBD-seq data are available from [GEO:GSE38679]. Details of DNA capture, preparation and sequencing can be found in Robinson *et al*. [26,37].

### Illumina HumanMethylation450 data for IMR-90, LNCaP and SssI DNA

IMR-90 and SssI DNA was processed by the Illumina HumanMethylation450 platform using the standard Illumina protocol. The raw (IDAT) and processed files are available at [49] under accession number [GEO:GSE54375]. The 450k array data for LNCaP cells originated from Robinson *et al*. [37] and can be found at [49] under accession number [GEO:GSE34340].

### Methyl binding domain sequencing and methylated DNA immunoprecipitation sequencing for comparing data from different methylation kits

For comparing data obtained by different methylation kits, we captured methylated DNA from IMR-90 and SssI DNA as follows. Genomic DNA was sheared to 150 to 200 bp using the Covaris S220 sonicator. MBDs were captured using the MethylMiner Methylated DNA Enrichment Kit (Invitrogen, Carlsbad, USA) and the MethylCap Kit (Diagenode, Liege, Belgium) following the manufacturers’ recommended protocols. The bound fractions were eluted at 500 mM and 1 M NaCl for MethylMiner and with buffers at different salt concentrations (low, medium, high) for MethylCap. Sequencing libraries were prepared with the SOLiD Fragment Library Construction Kit (Applied Biosystems, Foster City, USA). MeDIP-seq methylation immunocapture and library preparation were performed using the MeDIP Kit (Active Motif, Carlsbad, USA) following the manufacturer’s recommended protocol.

### Calculation of CpG density

CpG density is defined to be a weighted count of CpG sites in a predefined region. We used the function cpgDensityCalc provided by the R-package Repitools[50] to get bin-specific CpG density estimates using a linear weighting function and a window size of 700 bp (since we expect fragments around 300 bp).

### Calculation of mappability

Using Bowtie, all possible 36-bp reads of the genome were mapped back against the hg18 reference, with no mismatches. At each base, a read can either unambiguously map or not. A mappability estimate gives the proportion of reads that can be mapped to a specific regions. To get bin-specific mappability estimates, we used the function mappabilityCalc in the Repitools package [50]. In our analysis, a window of 500 bp was used (250 bp upstream and downstream from the center from each 100-bp bin) and the percentage of mappable bases was computed. For the methylation kits analysis we used mappability estimates for hg19 provided by ENCODE on [51], from which we derived a weighted mean based on the window size. Analogously, we used [52] for the Bock data analysis.

### Derivation of region-specific methylation estimates from whole genome bisulfite sequencing

In the Lister *et al*. IMR-90 WGBS data [40], the number of reads *rj* + and *rj*- overlaying a cytosine *j* in the positive (+) and negative strand (-), respectively, is available. Furthermore, the number of these reads, $m_{j}^{+}$ and $m_{j}^{-}$, that contain a methylated cytosine, is known. A single-base methylation estimate can be obtained as $\left(m_{j}^{+}+m_{j}^{-}\right)/\left(r_{j}^{+}+r_{j}^{-}\right)$. To get a bin-specific methylation estimate, all cytosines lying within a bin of interest  are taken into account:

$$\mu_{ℬ}=\frac{\underset{j\in ℬ}{\sum }\left(m_{j}^{+}+m_{j}^{-}\right)}{\underset{j\in ℬ}{\sum }\left(r_{j}^{+}+r_{j}^{-}\right)}$$

Here, $\underset{j\in ℬ}{\sum }\left(r_{j}^{+}+r_{j}^{-}\right)$ is termed the depth.

### Derivation of region-specific methylation estimates from 450K arrays

First, the Illumina HumanMethylation450 methylation array was preprocessed using the default parameters of the minfi package [53], version 1.3.3. For each sample, a vector of *beta values*, one for each targeted CpG site representing methylation estimates, is produced. To obtain (100 bp) bin-specific methylation profiles, we average beta values from all CpG sites within 100 bp (upstream and downstream; total window of 200 bp) from the center of our 100-bp bins.

### Derivation of region-specific methylation estimates from reduced representation bisulfite sequencing data

For the Bock data analysis, RRBS data were available from [43], which we consider to be the gold standard. Both the number of reads that overlay a cytosine (T) and the number of cytosines that stay a cytosine (M), i.e. are methylated, are given. Note that for one CpG site only information from one strand is available. To get smooth methylation estimates, we used 150-bp bins (overlapping by 100 bp). The methylation level for one 150-bp bin *i* was derived as:

$$m_{i}=\frac{\sum M_{\in i}}{\sum T_{\in i}}$$

That means using information for all CpG sites that fall into bin *i*.

### Determining the normalizing offset

The composition of a library influences the resulting read densities [54]. For example, the SssI control is a more diverse set of DNA fragments since it captures the vast majority of CpG-rich regions in the genome. Therefore, if the total sequencing depth were to be fixed, one would expect a relative undersampling of regions in SssI, compared to a sample of interest that is presumably largely unmethylated. To adjust the modeled mean (in the Poisson model) for these composition effects, we estimate a normalizing factor *f* that accounts simultaneously for overall sequencing depth and composition. Additional file 2: Figure S2 shows an *M* (log-ratio) versus *A* (average-log-count) plot for 50,000 randomly chosen (100 bp) bins for IMR-90 compared to the fully methylated control. A clear offset from zero is visible, since the distribution of *M* values is skewed in the negative direction. The normalization offset is estimated as $f=2^{\text{median}(M_{A>q})}$, with *q* corresponding to a high quantile of *A* (here, 0.998; more than 35,000 points). In cancer samples where CNV is common, the normalization factor *f* is calculated from bins that originate from the most prominent copy number state (e.g., ccn = 4 in LNCaP cells).

### Estimation of copy number

Copy numbers were estimated from Affymetrix SNP6.0 genotyping array data by PICNIC [55], using default parameters. PICNIC is an algorithm based on a hidden Markov model to produce absolute allelic copy number segmentation.

### BayMeth methodology

BayMeth’s processing can roughly be divided into two steps: 

1. An empirical Bayes procedure to derive sensible prior parameters for all parameters in the model.

2. The analytical derivation of the posterior marginal distribution, posterior expectation and variance for the methylation levels. Credible intervals are derived numerically from the posterior marginal distribution.

The details for both steps are provided in Additional file 1. In practice BayMeth can be used almost as a black box within the Bioconductor package Repitools[50].

### Batman specification

Batman is an algorithm implemented in Java and run from the command prompt. The original Batman can be downloaded from [56]. We used an unreleased version, 20090617, received directly from Thomas Down, which had MeDIP-seq-specific enhancements. The commands used to run Batman are given on the supplementary website [57].

### MEDIPS specification

We used R-Bioconductor MEDIPS version 1.4.0. The detailed command sequence is given on the supplementary website [57]. MEDIPS returns methylation estimates in the range from zero to 1,000, which we rescaled to the interval [ 0,1]. In our comparison, we used the absolute methylation score provided by MEDIPS.

### BALM specification

BALM is an algorithm implemented in C and C++ and run from the command prompt. The original BALM can be downloaded from [58]. We used version 1.01. The detailed command sequence is given on the supplementary website [57]. BALM returns a vector of methylation estimates, one for each targeted CpG site. To obtain (100 bp) bin-specific methylation profiles, we averaged the methylation estimates from all CpG sites within 100 bp (upstream and downstream; total window of 200 bp) from the center of our 100-bp bins. For the IMR-90 dataset, BALM was run without an input control. To assess the effect of the missing input control, we ran BALM using a sample from a normal human prostate epithelial cell line (PrEC) as input control, which gave almost identical performance results.

### Software

BayMeth is fully integrated into the R package Repitools and available from the Bioconductor project. Data (semi-processed), R code for all figures and analyses are provided on [57].

## Abbreviations

bp: base pair; CNV: copy number variation; DNAme: DNA methylation; HPD: highest posterior density; LNCaP: human prostate carcinoma; MBD: methyl binding domain; MeDIP: methylated DNA immunoprecipitation; MSE: mean of squared differences; RRBS: reduced representation bisulfite sequencing; seq: sequencing; WGBS: whole genome bisulfite sequencing.

## Competing interests

The authors declare that they have no competing interests.

## Authors’ contributions

The statistical approach was conceived and developed by AR and MDR, with biological and technical insight from ALS, CS, MM and SJC. Implementation and data analyses were done by AR with contributions from MDR. Data was collected by JZS, ALS, NM, CM and MM. AR and MDR wrote the manuscript with input from all authors. All authors read and approved the final manuscript.

## Supplementary Material

Additional file 1 **Statistical details of BayMeth.** This document describes the BayMeth methodology. Two different prior distributions for the methylation level are presented, namely, a mixture of beta distributions, and a mixture of a point mass at zero, a beta distribution and a point mass at one (Dirac-beta-Dirac prior). An empirical Bayes procedure is used to derive prior parameters. The analytical derivation of the posterior marginal distribution and parameter estimation is described for both priors. We outline the derivations for the standard BayMeth version, i.e. taking advantage of SssI information, and for the SssI-free version. Click here for file

Additional file 2 **Supplementary figures and tables.** This document contains six supplementary figures and one supplementary table. Detailed descriptions are provided within the file. Click here for file

Additional file 3 **BayMeth analysis of Bock data.** This document outlines all data preparation steps performed and presents detailed R code for the BayMeth analysis conducted using the Bioconductor package Repitools. Click here for file
