# Supplementary material for: BayMeth: improved DNA methylation quantification for affinity capture sequencing data using a flexible Bayesian approach
Source: Genome Biol. 2014 Feb 11;15(2):R35. doi: 10.1186/gb-2014-15-2-r35 (PMC4053803; doi:10.1186/gb-2014-15-2-r35)
Supplement: Additional file 1 — Statistical details of BayMeth. This document describes the BayMeth methodology. Two different prior distributions for the methylation level are presented, namely, a mixture of beta distributions, and a mixture of a point mass at zero, a beta distribution and a point mass at one (Dirac-beta-Dirac prior). An empirical Bayes procedure is used to derive prior parameters. The analytical derivation of the posterior marginal distribution and parameter estimation is described for both priors. We outline the derivations for the standard BayMeth version, i.e. taking advantage of SssI information, and for the SssI-free version. [file gb-2014-15-2-r35-S1.pdf]

Additional file 1 — Statistical details of BayMeth

Andrea Riebler[1,2,3,*], Mirco Menigatti[4], Jenny Z. Song[5], Aaron L. Statham[5] , Clare Stirzaker[5,6], Nadiya Mahmud[7], Charles A. Mein[7], Susan J. Clark[5,6] , Mark D. Robinson[1,8,*]

[1]Institute of Molecular Life Sciences, University of Zurich, Winterthurerstrasse 190, CH-8057 Zurich, Switzerland
[2]Institute of Social- and Preventive Medicine, University of Zurich, Hirschengraben 84, CH-8001 Zurich, Switzerland
[3]Department of Mathematical Sciences, Norwegian University of Science and Technology, N-7491 Trondheim, Norway
[4]Institute of Molecular Cancer Research, University of Zurich, Winterthurerstrasse 190, CH-8057 Zurich, Switzerland
[5]Epigenetics Laboratory, Cancer Research Program, Garvan Institute of Medical Research, Sydney 2010, New South Wales, Australia
[6]St Vincent's Clinical School, University of NSW, Sydney 2052, NSW, Australia
[7]Genome Centre, Barts and the London, Queen Mary, University of London, Charterhouse Square, London EC1M 6BQ, United Kingdom
[8]SIB Swiss Institute of Bioinformatics, University of Zurich, Zurich, Switzerland

Email: Andrea Riebler*- andrea.riebler@math.ntnu.no; Mirco Menigatti - menigatti@imcr.uzh.ch; Jenny Z. Song - j.song@garvan.org.au; Aaron L. Statham- a.statham@garvan.org.au; Clare Stirzaker - c.stirzaker@garvan.org.au; Nadiya Mahmud - n.mahmud@qmul.ac.uk; Charles A. Mein - c.a.mein@qmul.ac.uk; Susan J. Clark - s.clark@garvan.org.au; Mark D. Robinson*- mark.robinson@imls.uzh.ch;

*Corresponding author

The methodology of BayMeth is roughly divided into two steps: 1) An empirical Bayes procedure to derive parameters for the prior distributions of all parameters in the model. 2) The analytical derivation of the posterior marginal distribution, posterior expectation and variance for the methylation levels. Credible intervals are derived numerically from the posterior marginal distribution. Recall the model formulation provided in the main text:

$$
\begin{aligned}
y_{iS}|\mu_i, \lambda_i &\sim \text{Poisson}\left(f \times \frac{\text{cn}_i}{\text{ccn}} \times \mu_i \times \lambda_i\right), \text{ and} \\
y_{iC}|\lambda_i &\sim \text{Poisson}(\lambda_i),
\end{aligned}
$$

Prior specification

For λ_i we assume a gamma prior distribution with parameters α and β:

$$
\lambda_i \mid \alpha, \beta = \frac{\beta^\alpha}{\Gamma(\alpha)} \lambda_i^{\alpha-1} \exp(-\beta\lambda_i), \lambda_i > 0, \alpha, \beta > 0.
$$

The methylation level μ_i has support from zero to one. We consider two groups of prior distributions:

- a mixture of beta distributions, i.e., $\mu_i \sim \sum_{m=1}^{M} w_m \,\text{Be}(a_m, b_m)$, where in its simplest form $M = 1$. (The default configuration of BayMeth is $M = 1$ and $(a = a_m = b = b_m = 1)$, i.e., a uniform distribution from zero to one.)

- a mixture of a point mass at zero, a beta distribution and a point mass at one. We call this the Dirac-Beta-Dirac (DBD) prior distribution, which has the density

$$p(\mu_i) = w_0\delta_0 + w_1 \, \mathrm{Be}(\mu_i; a, b) + w_2\delta_1, \tag{1}$$

where

$$\delta_0 = \begin{cases} 0 & \text{if } \mu_i \neq 0 \\ 1 & \text{if } \mu_i = 0 \end{cases}, \qquad \delta_1 = \begin{cases} 0 & \text{if } \mu_i \neq 1 \\ 1 & \text{if } \mu_i = 1 \end{cases}$$

and $w_0 + w_1 + w_2 = 1$.

Marginal distribution

The empirical Bayes approach is based on the maximization of the marginal distribution. For ease of readability let $E = f \times \frac{\mathrm{cn}_i}{\mathrm{ccn}}$. The joint marginal distribution of y_{iS}, y_{iC} results as:

$$\begin{aligned}
p(y_{iS}, y_{iC}) &= \int\int p(y_{iS}|\mu_i, \lambda_i) p(y_{iC}|\lambda_i) p(\lambda_i) p(\mu_i) d\lambda_i d\mu_i \\
&= \int_0^1 p(\mu_i) \left[\int_0^\infty p(y_{iS}|\mu_i, \lambda_i) p(y_{iC}|\lambda_i) p(\lambda_i) d\lambda_i \right] d\mu_i \\
&= \int_0^1 p(\mu_i) \left[\int_0^\infty \frac{(E\mu_i\lambda_i)^{y_{iS}} \lambda_i^{y_{iC}}}{y_{iS}! y_{iC}!} \exp\left(-E\mu_i\lambda_i\right) \times \exp(-\lambda_i) \times \frac{\beta^\alpha}{\Gamma(\alpha)} \lambda_i^{\alpha-1} \exp(-\beta\lambda_i) d\lambda_i \right] d\mu_i \\
&= \int_0^1 p(\mu_i) \left[\frac{(E\mu_i)^{y_{iS}}}{y_{iS}! y_{iC}!} \frac{\beta^\alpha}{\Gamma(\alpha)} \int_0^\infty \lambda_i^{y_{iS}+y_{iC}+\alpha-1} \exp\left(-(E\mu_i + 1 + \beta)\lambda_i\right) d\lambda_i \right] d\mu_i \\
&= \int_0^1 p(\mu_i) \left[\frac{(E\mu_i)^{y_{iS}}}{y_{iS}! y_{iC}!} \frac{\beta^\alpha}{\Gamma(\alpha)} \frac{\Gamma(y_{iS} + y_{iC} + \alpha)}{(E\mu_i + 1 + \beta)^{y_{iS}+y_{iC}+\alpha}} \right] d\mu_i \\
&= \frac{E^{y_{iS}}}{y_{iS}! y_{iC}!} \frac{\beta^\alpha}{\Gamma(\alpha)} \Gamma(y_{iS} + y_{iC} + \alpha) \int_0^1 p(\mu_i) \frac{\mu_i^{y_{iS}}}{(E\mu_i + 1 + \beta)^{y_{iS}+y_{iC}+\alpha}} d\mu_i
\end{aligned}$$

What is left, is to choose a prior for μ_i, that means to specify $p(\mu_i)$.

(Mixture of) beta distribution for the methylation level

Consider the simple case where the number of mixture components is one, so that $\mu_i \sim \text{Be}(a, b)$, i.e. $p(\mu_i) = \frac{\Gamma(a+b)}{\Gamma(a)\Gamma(b)}\mu_i^{a-1}(1-\mu_i)^{b-1}$, $a, b > 0$. (For a uniform distribution $a = b = 1$). Then

$$\begin{aligned}
p(y_{iS}, y_{iC}) &= \frac{\Gamma(y_{iS}+y_{iC}+\alpha)}{\Gamma(\alpha)y_{iS}!y_{iC}!}\frac{\Gamma(a+b)}{\Gamma(a)\Gamma(b)}E^{y_{iS}}\beta^{\alpha}\int_0^1 \frac{\mu_i^{y_{iS}+a-1}(1-\mu_i)^{b-1}}{(E\mu_i+1+\beta)^{y_{iS}+y_{iC}+\alpha}}d\mu_i \\
&= \frac{\Gamma(y_{iS}+y_{iC}+\alpha)}{\Gamma(\alpha)y_{iS}!y_{iC}!}\frac{\Gamma(a+b)}{\Gamma(a)\Gamma(b)}E^{y_{iS}}\frac{\beta^{\alpha}}{(E+1+\beta)^{y_{iS}+y_{iC}+\alpha}}\int_0^1 \frac{\mu_i^{y_{iS}+a-1}(1-\mu_i)^{b-1}}{\frac{(E\mu_i+1+\beta)^{y_{iS}+y_{iC}+\alpha}}{(E+1+\beta)^{y_{iS}+y_{iC}+\alpha}}}d\mu_i \\
&= \frac{\Gamma(y_{iS}+y_{iC}+\alpha)}{\Gamma(\alpha)y_{iS}!y_{iC}!}\frac{\Gamma(a+b)}{\Gamma(a)\Gamma(b)}E^{y_{iS}}\frac{\beta^{\alpha}}{(\beta+1+E)^{y_{iS}+y_{iC}+\alpha}}\int_0^1 \frac{\mu_i^{y_{iS}+a-1}(1-\mu_i)^{b-1}}{\left(1-\frac{E}{E+1+\beta}\cdot(1-\mu_i)\right)^{y_{iS}+y_{iC}+\alpha}}d\mu_i \\
&\overset{\star}{=} \frac{\Gamma(y_{iS}+y_{iC}+\alpha)}{\Gamma(\alpha)y_{iS}!y_{iC}!}\frac{\Gamma(a+b)}{\Gamma(a)\Gamma(b)}E^{y_{iS}}\frac{\beta^{\alpha}}{(\beta+1+E)^{y_{iS}+y_{iC}+\alpha}}\int_0^1 \frac{(1-t_i)^{y_{iS}+a-1}t_i^{b-1}}{\left(1-\frac{E}{E+1+\beta}\cdot t_i\right)^{y_{iS}+y_{iC}+\alpha}}dt_i \\
&= \frac{\Gamma(y_{iS}+y_{iC}+\alpha)}{\Gamma(\alpha)y_{iS}!y_{iC}!}\left(\frac{\beta}{\beta+1+E}\right)^{\alpha}\left(\frac{E}{\beta+1+E}\right)^{y_{iS}}\left(\frac{1}{\beta+1+E}\right)^{y_{iC}}\frac{\Gamma(a+b)\Gamma(y_{iS}+a)}{\Gamma(a)\Gamma(y_{iS}+a+b)}\times \\
&\qquad {}_2F_1\left(y_{iS}+y_{iC}+\alpha, b; y_{iS}+a+b; \frac{E}{\beta+1+E}\right).
\end{aligned} \tag{2}$$

In the step marked with * we substituted $(1-\mu_i)$ with t_i, where $dt_i = -d\mu_i$, to get the desired form of the Gauss hypergeometric function (the limits of the integral stay thereby unchanged), which is defined by:

$$_2F_1(a, b; c; z) = \frac{\Gamma(c)}{\Gamma(b)\Gamma(c-b)}\int_0^1 t^{b-1}(1-t)^{c-b-1}(1-zt)^{-a}dt, \quad c > b > 0$$

where $|z| < 1$ is the radius of convergence [1, see page 558]. (Note, $|z| = |E/(\beta+1+E)| < 1$ in (2), so that convergence is granted). Model (2) is similar to the beta binomial (BB)/negative binomial (NB) model derived in [2] and [3].

Using a mixture of M beta distributions as prior distribution for μ_i, i.e. $\mu_i \sim \sum_{m=1}^M w_m \,\text{Be}(a_m, b_m)$, where $0 \le w_m \le 1$, for all $m = 1, \ldots, M$, and $\sum_{m=1}^M w_m = 1$ we get:

$$p(y_{iS}, y_{iC}) = \frac{\Gamma(y_{iS}+y_{iC}+\alpha)}{\Gamma(\alpha)y_{iS}!y_{iC}!}\left(\frac{\beta}{\beta+1+E}\right)^{\alpha}\left(\frac{E}{\beta+1+E}\right)^{y_{iS}}\left(\frac{1}{\beta+1+E}\right)^{y_{iC}} \times W$$

with

$$W = \sum_{m=1}^M\left[w_m \cdot \frac{\Gamma(a_m+b_m)\Gamma(y_{iS}+a_m)}{\Gamma(a_m)\Gamma(y_{iS}+a_m+b_m)} \times \, {}_2F_1\left(y_{iS}+y_{iC}+\alpha, b_m; y_{iS}+a_m+b_m; \frac{E}{\beta+1+E}\right)\right].$$

Of note, ignoring the SssI information the marginal distribution changes to:

$$\begin{aligned}
p(y_{iS}) &= \int\int p(y_{iS}|\mu_i, \lambda_i)p(\lambda_i)p(\mu_i)d\lambda_i d\mu_i \\
&= \frac{\Gamma(y_{iS}+\alpha)}{\Gamma(\alpha)y_{iS}!}\left(\frac{\beta}{\beta+E}\right)^{\alpha}\left(\frac{E}{\beta+E}\right)^{y_{iS}} \times W
\end{aligned}$$

with

$$W = \sum_{m=1}^{M} \left[w_m \cdot \frac{\Gamma(a_m+b_m)\Gamma(y_{iS}+a_m)}{\Gamma(a_m)\Gamma(y_{iS}+a_m+b_m)} \times {}_2F_1 \left(y_{iS}+\alpha, b_m; y_{iS}+a_m+b_m; \frac{E}{\beta+E} \right) \right]. \tag{3}$$

Dirac-beta-Dirac distribution for the methylation level

If we consider instead of a mixture beta distribution, the DBD prior as given in Equation (1), we get the following marginal distribution:

$$p(y_{iS}, y_{iC}) = \frac{\Gamma(y_{iS}+y_{iC}+\alpha)}{\Gamma(\alpha) y_{iS}! y_{iC}!} \left(\frac{\beta}{\beta+1+E} \right)^{\alpha} \left(\frac{E}{\beta+1+E} \right)^{y_{iS}} \left(\frac{1}{\beta+1+E} \right)^{y_{iC}} \times W$$

with

$$W = w_2 + w_1 \cdot \frac{\Gamma(a+b)\Gamma(y_{iS}+a)}{\Gamma(a)\Gamma(y_{iS}+a+b)} \times {}_2F_1 \left(y_{iS}+y_{iC}+\alpha, b; y_{iS}+a+b; \frac{E}{\beta+E+1} \right) \Bigg].$$

Ignoring the SssI information this marginal distribution changes to:

$$p(y_{iS}) = \frac{\Gamma(y_{iS}+\alpha)}{\Gamma(\alpha) y_{iS}!} \left(\frac{\beta}{\beta+E} \right)^{\alpha} \left(\frac{E}{\beta+E} \right)^{y_{iS}} \times W$$

with

$$W = w_2 + w_1 \cdot \frac{\Gamma(a+b)\Gamma(y_{iS}+a)}{\Gamma(a)\Gamma(y_{iS}+a+b)} \times {}_2F_1 \left(y_{iS}+\alpha, b; y_{iS}+a+b; \frac{E}{\beta+E} \right) \Bigg].$$

Parameter estimation

Independent of the prior choice for μ_i, we have to determine parameters α and β of the gamma prior distribution for λ. The default BayMeth assumes a uniform prior for μ_i, i.e. $M = 1$ and $\mu_i \sim \text{Be}(a = 1, b = 1)$, and that SssI information is taken into account, therefore α and β are the only parameters to determine. Under the empirical Bayes approach, the parameters α and β of Equation (2) can be estimated using maximum likelihood. The parameters are thereby determined in a CpG-density-dependent manner. Each 100bp bin is classified based on its CpG-density into one of $K = 100$ non-overlapping CpG-density classes: $\mathcal{C}_1, \ldots, \mathcal{C}_K$. The class size $|\mathcal{C}_k|$, i.e. the number of 100bp bins in class k, is denoted by n_k. We derive for each class separately the set of prior parameters using empirical Bayes leading finally to K parameter sets. The corresponding log likelihood function for class k is then given by

$$l(\alpha^{(k)}, \beta^{(k)} | \mathbf{y}_1^{(k)}, \mathbf{y}_2^{(k)}) = \sum_{j=1}^{n_k} \log(p(y_{j1}^{(k)}, y_{j2}^{(k)} | \alpha^{(k)}, \beta^{(k)}). \tag{4}$$

Here $\mathbf{y}_S^{(k)} = (y_{1S}^{(k)}, \ldots, y_{n_k S}^{(k)})$ and $\mathbf{y}_C^{(k)} = (y_{1C}^{(k)}, \ldots, y_{n_k C}^{(k)})$ denote the read counts of the bins contained in class $\mathcal{C}_k$. Further $\alpha^{(k)}$, $\beta^{(k)}$ denote the parameters for CpG-density class k. In Equation (4), we assume that genomic regions are independent. For a discussion of this assumption, see the Discussion Section of the main paper. Considering a different prior distribution for μ_i the empirical Bayes approach extends to the additional parameters appearing in the prior. They will be also estimated in a CpG dependent manner. However, one should avoid including too many parameters as this complicates the empirical Bayes procedure and makes it more difficult to find the best parameters. In the case of the DBD prior distribution we fixed the weights to $w_0 = 0.1$, $w_1 = 0.8$, $w_2 = 0.1$ and only estimated the parameters a and b.

Derivation of the posterior marginal distribution

Using a beta mixture prior for the methylation level

Our main interest lies in the marginal posterior distribution of the methylation level μ_i

$$p(\mu_i | y_{iS}, y_{iC}) = \int_0^\infty p(\lambda_i, \mu_i | y_{iS}, y_{iC}) d\lambda_i,$$

where

$$\begin{aligned} p(\lambda_i, \mu_i | y_{iS}, y_{iC}) &= \frac{p(y_{iS}, y_{iC} | \lambda_i, \mu_i) p(\lambda_i, \mu_i)}{p(y_{iS}, y_{iC})} \\ &\overset{\text{cond.indep}}{=} \frac{p(y_{iS} | \lambda_i, \mu_i) p(y_{iC} | \lambda_i) p(\lambda_i) p(\mu_i)}{p(y_{iS}, y_{iC})} \\ &= \frac{\lambda_i^{y_{iS} + y_{iC} + \alpha - 1} \exp(-(E\mu_i + 1 + \beta)\lambda_i)(\beta + 1 + E)^{\alpha + y_{iS} + y_{iC}} p(\mu_i) \mu_i^{y_{iS}}}{\Gamma(y_{iS} + y_{iC} + \alpha) \times W}. \end{aligned}$$

Here, W is as given in Equation (3), and α and β are the parameters for the gamma prior distribution for λ_i as determined by empirical Bayes (see above) for the CpG-density class to which bin i belongs.

Thus:

$$\begin{aligned} p(\mu_i | y_{iS}, y_{iC}) &= \frac{\mu_i^{y_{iS}} p(\mu_i)(\beta + 1 + E)^{\alpha + y_{iS} + y_{iC}}}{\Gamma(y_{iS} + y_{iC} + \alpha) \times W} \int_0^\infty \lambda_i^{y_{iS} + y_{iC} + \alpha - 1} \exp(-(E\mu_i + 1 + \beta)\lambda_i) d\lambda_i \\ &= \frac{\mu_i^{y_{iS}} p(\mu_i)}{W} \left(1 - \frac{E(1 - \mu_i)}{\beta + 1 + E}\right)^{-(\alpha + y_{iS} + y_{iC})}. \end{aligned}$$

The mean of the marginal posterior of μ_i is given by:

$$\mathsf{E}(\mu_i | y_{iS}, y_{iC}) = \frac{A}{W}$$

with

$$A = \sum_{m=1}^{M} \left[w_m \cdot \frac{\Gamma(a_m + b_m)\Gamma(y_{iS} + a_m + 1)}{\Gamma(a_n)\Gamma(y_{iS} + a_m + b_m + 1)} \times {}_2F_1\left(y_{iS} + y_{iC} + \alpha, b_m; y_{iS} + a_m + b_m + 1; \frac{E}{\beta + 1 + E}\right)\right].$$

Proof.

$$\begin{aligned}\mathsf{E}(\mu_i|y_{iS},y_{iC}) &= \int_0^1 \mu_i p(\mu_i|y_{iS},y_{iC})d\mu_i \\ &= \frac{1}{W}\sum_{m=1}^{M}\left[\int_0^1 \frac{w_m \frac{\Gamma(a_m+b_m)}{\Gamma(a_m)\Gamma(b_m)}\mu_i^{a_m+y_{iS}}(1-\mu_i)^{b_m-1}}{\left(1-\frac{E(1-\mu_i)}{\beta+1+E}\right)^{\alpha+y_{iS}+y_{iC}}}d\mu_i\right],\end{aligned}$$

where each integral can again be written in terms of the Gauss hypergeometric function:

$$\begin{aligned}&\int_0^1 \frac{w_m \frac{\Gamma(a_m+b_m)}{\Gamma(a_m)\Gamma(b_m)}\mu_i^{a_m+y_{iS}}(1-\mu_i)^{b_m-1}}{\left(1-\frac{E(1-\mu_i)}{\beta+1+E}\right)^{\alpha+y_{iS}+y_{iC}}}d\mu_i \\ &= \frac{w_m\Gamma(a_m+b_m)}{\Gamma(a_m)\Gamma(b_m)}\int_0^1 \frac{(1-t_i)^{a_m+y_{iS}}t_i^{b_m-1}}{\left(1-\frac{E}{\beta+1+E}t_i\right)^{\alpha+y_{iS}+y_{iC}}}dt_i \\ &= \frac{w_m\Gamma(a_m+b_m)}{\Gamma(a_m)\Gamma(b_m)}\frac{\Gamma(b_m)\Gamma(y_{iS}+a_m+1)}{\Gamma(y_{iS}+a_m+b_m+1)}\,{}_2F_1\left(y_{iS}+y_{iC}+\alpha, b_m; y_{iS}+a_m+b_m+1; \frac{E}{\beta+1+E}\right) \\ &= \frac{w_m\Gamma(a_m+b_m)\Gamma(y_{iS}+a_m+1)}{\Gamma(a_m)\Gamma(y_{iS}+a_m+b_m+1)}\,{}_2F_1\left(y_{iS}+y_{iC}+\alpha, b_m; y_{iS}+a_m+b_m+1; \frac{E}{\beta+1+E}\right).\end{aligned}$$

□

The variance of the marginal posterior distribution of μ_i can be computed using the computational formula for the variance $\text{Var}(\mu_i|y_{iS},y_{iC}) = \mathsf{E}(\mu_i^2|y_{iS},y_{iC}) - (\mathsf{E}(\mu_i|y_{iS},y_{iC}))^2$, where

$$\mathsf{E}(\mu_i^2|y_{iS},y_{iC}) = \frac{B}{W}$$

with

$$B = \sum_{m=1}^{M}\left[w_m \cdot \frac{\Gamma(a_m+b_m)\Gamma(y_{iS}+a_n+2)}{\Gamma(a_m)\Gamma(y_{iS}+a_m+b_m+2)} \times {}_2F_1\left(y_{iS}+y_{iC}+\alpha, b_m; y_{iS}+a_m+b_m+2; \frac{E}{\beta+1+E}\right)\right],$$

so that

$$\text{Var}(\mu_i|y_{iS},y_{iC}) = \frac{B}{W} - \left(\frac{A}{W}\right)^2.$$

Running BayMeth without a fully methylated control sample, we get

$$\mathsf{E}(\mu_i|y_{iS}) = \frac{A}{W}\ \text{Var}(\mu_i|y_{iS}) = \frac{B}{W} - \left(\frac{A}{W}\right)^2.$$

A, B and W are:

$$A = \sum_{m=1}^{M} \left[w_m \cdot \frac{\Gamma(a_m + b_m)\Gamma(y_{iS} + a_m + 1)}{\Gamma(a_m)\Gamma(y_{iS} + a_m + b_m + 1)} \times {}_2F_1 \left(y_{iS} + \alpha, b_m; y_{iS} + a_m + b_m + 1; \frac{E}{\beta + E} \right) \right],$$

$$B = \sum_{m=1}^{M} \left[w_m \cdot \frac{\Gamma(a_m + b_m)\Gamma(y_{iS} + a_m + 2)}{\Gamma(a_m)\Gamma(y_{iS} + a_m + b_m + 2)} \times {}_2F_1 \left(y_{iS} + \alpha, b_m; y_{iS} + a_m + b_m + 2; \frac{E}{\beta + E} \right) \right],$$

$$W = \sum_{m=1}^{M} \left[w_m \cdot \frac{\Gamma(a_m + b_m)\Gamma(y_{iS} + a_m)}{\Gamma(a_m)\Gamma(y_{iS} + a_m + b_m)} \times {}_2F_1 \left(y_{iS} + \alpha, b_m; y_{iS} + a_m + b_m; \frac{E}{\beta + E} \right) \right].$$

Using a DBD prior for the methylation level

The posterior mean and variance can be derived analogously to the previous section. Borrowing strength from a SssI sample, posterior mean and variance are given by:

$$\mathsf{E}(\mu_i | y_{iS}, y_{iC}) = \frac{A}{W}$$

$$\mathrm{Var}(\mu_i | y_{iS}, y_{iC}) = \frac{B}{W} - \left(\frac{A}{W} \right)^2.$$

with

$$A = w_2 + w_1 \cdot \frac{\Gamma(a + b)\Gamma(y_{iS} + a + 1)}{\Gamma(a)\Gamma(y_{iS} + a + b + 1)} \times {}_2F_1 \left(y_{iS} + y_{iC} + \alpha, b; y_{iS} + a + b + 1; \frac{E}{\beta + E + 1} \right) \Bigg],$$

$$B = w_2 + w_1 \cdot \frac{\Gamma(a + b)\Gamma(y_{iS} + a + 2)}{\Gamma(a)\Gamma(y_{iS} + a + b + 2)} \times {}_2F_1 \left(y_{iS} + y_{iC} + \alpha, b; y_{iS} + a + b + 2; \frac{E}{\beta + E + 1} \right) \Bigg],$$

$$W = w_2 + w_1 \cdot \frac{\Gamma(a + b)\Gamma(y_{iS} + a)}{\Gamma(a)\Gamma(y_{iS} + a + b)} \times {}_2F_1 \left(y_{iS} + y_{iC} + \alpha, b; y_{iS} + a + b; \frac{E}{\beta + E + 1} \right) \Bigg].$$

Assuming that no SssI sample is available, then

$$\mathsf{E}(\mu_i | y_{iS}) = \frac{A}{W}$$

$$\mathrm{Var}(\mu_i | y_{iS}) = \frac{B}{W} - \left(\frac{A}{W} \right)^2.$$

with

$$A = w_2 + w_1 \cdot \frac{\Gamma(a+b)\Gamma(y_{iS}+a+1)}{\Gamma(a)\Gamma(y_{iS}+a+b+1)} \times \ {}_2F_1\left(y_{iS}+\alpha, b; y_{iS}+a+b+1; \frac{E}{\beta+E}\right)\Bigg],$$

$$B = w_2 + w_1 \cdot \frac{\Gamma(a+b)\Gamma(y_{iS}+a+2)}{\Gamma(a)\Gamma(y_{iS}+a+b+2)} \times \ {}_2F_1\left(y_{iS}+\alpha, b; y_{iS}+a+b+2; \frac{E}{\beta+E}\right)\Bigg],$$

$$W = w_2 + w_1 \cdot \frac{\Gamma(a+b)\Gamma(y_{iS}+a)}{\Gamma(a)\Gamma(y_{iS}+a+b)} \times \ {}_2F_1\left(y_{iS}+\alpha, b; y_{iS}+a+b; \frac{E}{\beta+E}\right)\Bigg].$$

References

1. Abramowitz M, Stegun IA: *Handbook of Mathematical functions with Formulas, Graphs and Mathematical Tables*. New York: Dover Publications 1972.

2. Schmittlein DC, Bemmaor AC, Morrison DG: **Why does the NBD model work? Robustness in representing product purchases, brand purchases and imperfectly recorded purchases**. *Marketing Science* 1985, **4**(3):pp. 255–266.

3. Fader PS, Hardie BGS: **A note on modelling underreported Poisson counts**. *Journal of Applied Statistics* 2000, **27**(8):953–964.
