# Supplementary material for: BayMeth: improved DNA methylation quantification for affinity capture sequencing data using a flexible Bayesian approach
Source: Genome Biol. 2014 Feb 11;15(2):R35. doi: 10.1186/gb-2014-15-2-r35 (PMC4053803; doi:10.1186/gb-2014-15-2-r35)
Supplement: Additional file 2 — Supplementary figures and tables. This document contains six supplementary figures and one supplementary table. Detailed descriptions are provided within the file. [file gb-2014-15-2-r35-S2.pdf]

Additional file 2 — Supplementary figures and tables

Andrea Riebler[1,2,3,*], Mirco Menigatti[4], Jenny Z. Song[5], Aaron L. Statham[5] , Clare Stirzaker[5,6], Nadiya Mahmud[7], Charles A. Mein[7], Susan J. Clark[5,6] , Mark D. Robinson[1,8,*]

[1]Institute of Molecular Life Sciences, University of Zurich, Winterthurerstrasse 190, CH-8057 Zurich, Switzerland
[2]Institute of Social- and Preventive Medicine, University of Zurich, Hirschengraben 84, CH-8001 Zurich, Switzerland
[3]Department of Mathematical Sciences, Norwegian University of Science and Technology, N-7491 Trondheim, Norway
[4]Institute of Molecular Cancer Research, University of Zurich, Winterthurerstrasse 190, CH-8057 Zurich, Switzerland
[5]Epigenetics Laboratory, Cancer Research Program, Garvan Institute of Medical Research, Sydney 2010, New South Wales, Australia
[6]St Vincent's Clinical School, University of NSW, Sydney 2052, NSW, Australia
[7]Genome Centre, Barts and the London, Queen Mary, University of London, Charterhouse Square, London EC1M 6BQ, United Kingdom
[8]SIB Swiss Institute of Bioinformatics, University of Zurich, Zurich, Switzerland

Email: Andrea Riebler*- andrea.riebler@math.ntnu.no; Mirco Menigatti - menigatti@imcr.uzh.ch; Jenny Z. Song - j.song@garvan.org.au; Aaron L. Statham- a.statham@garvan.org.au; Clare Stirzaker - c.stirzaker@garvan.org.au; Nadiya Mahmud - n.mahmud@qmul.ac.uk; Charles A. Mein - c.a.mein@qmul.ac.uk; Susan J. Clark - s.clark@garvan.org.au; Mark D. Robinson*- mark.robinson@imls.uzh.ch;

*Corresponding author

Supplementary Figures

Figure S1 - CpG-density stratified by CpG island status

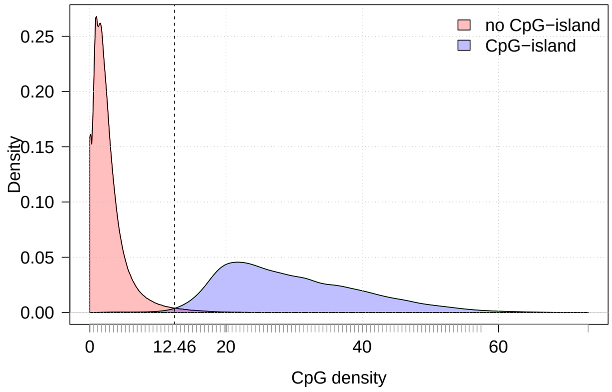

Genome-wide CpG-density for bins with a mappability larger than 75% stratified by CpG island status as extracted from the cpgIslandExt-table of the UCSC genome browser. The vertical line marks the intersection of both densities. The grey tick-marks along the x-axis illustrate the CpG-density classes used for the empirical Bayes approach in the IMR-90 application.

Figure S2 - Normalizing offset for IMR-90

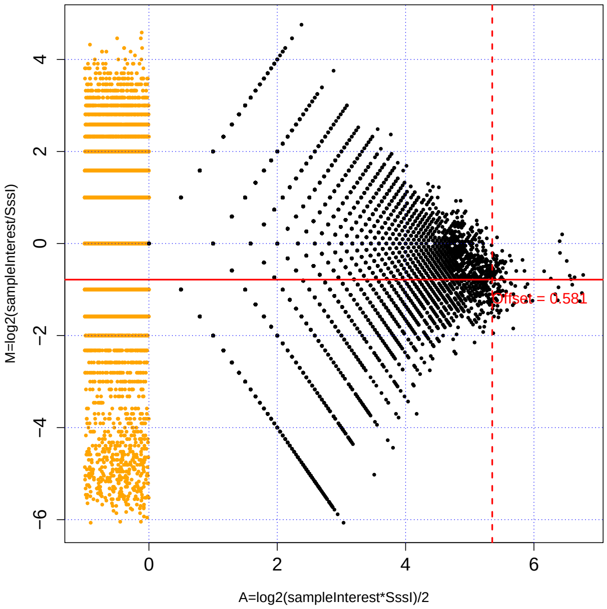

Log-fold change (M) versus log-concentration (A) illustrated for 50000 randomly chosen bins. The red dotted line shows the 0.998 quantile q of A determined from all bins. The red straight line shows the estimated normalization offset $f = 2^{median(M_{A>q})}$. A 'smear' of yellow points at a low A value represents counts that are low in either of the two samples.

Figure S3 - Copy number frequencies for LNCaP

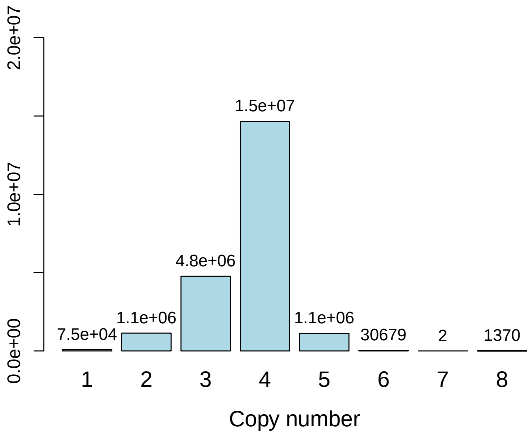

Copy number frequencies in LNCaP for 100bp-bins with a mappability larger than 0.75.

Figure S4 - Read depth of LNCaP MBD-seq by copy number

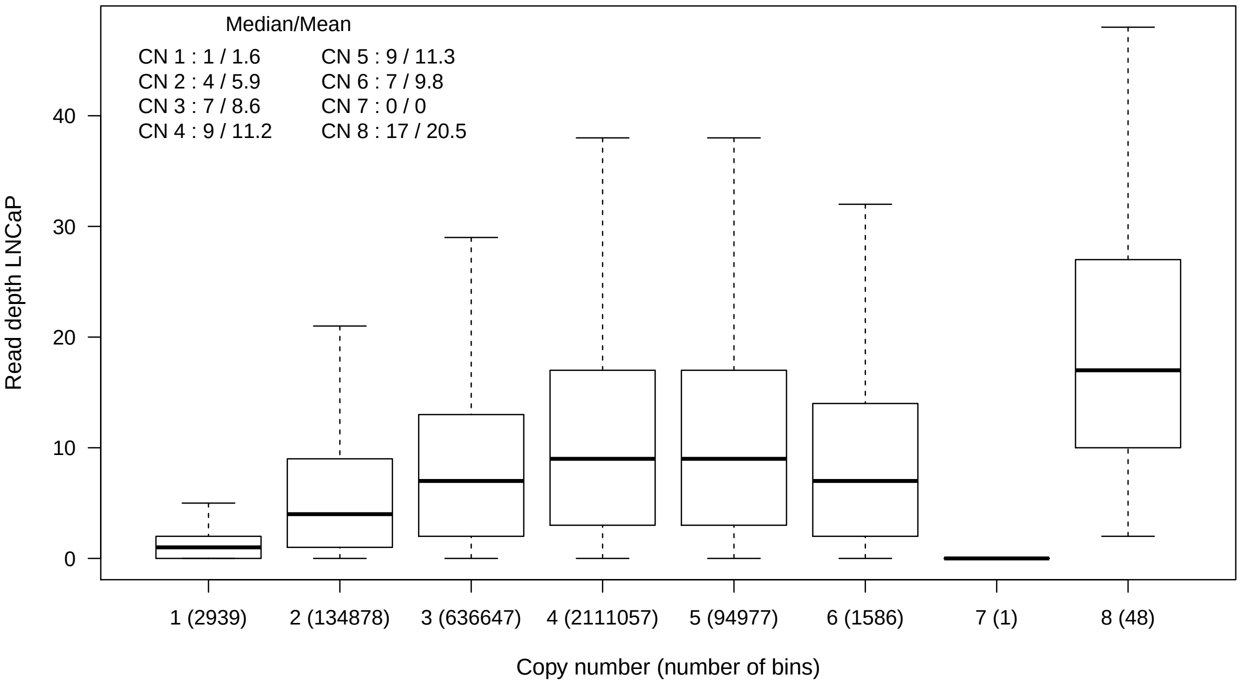

Read depth stratified by copy number is shown for 100bp-bins with a mappability larger than 0.75 and with a SssI depth larger than four. Median and mean read depth are given per copy number state.

Figure S5 - Varying normalizing offsets between methylation kits

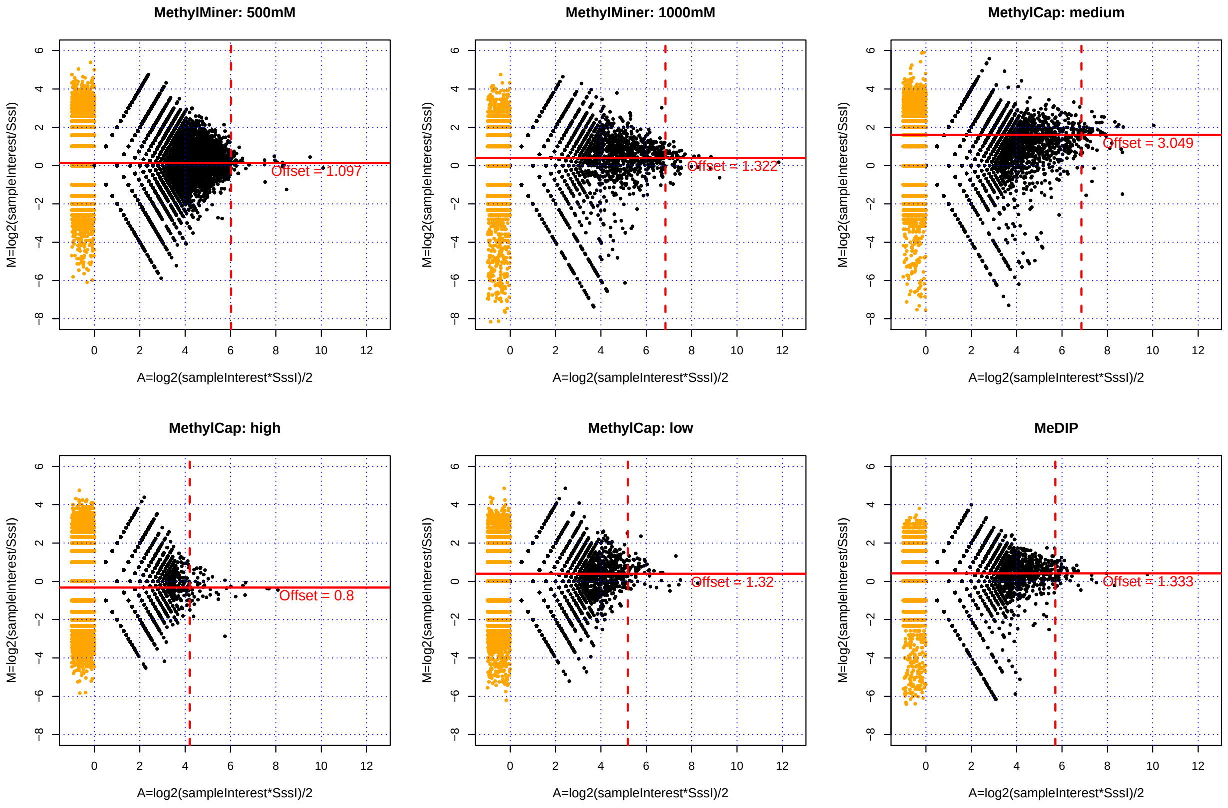

Log-fold change (M) versus log-concentration (A) illustrated for 25000 randomly chosen bins for IMR-90 data derived using different methylation kits. The red dotted line shows the 0.998 quantile q of A determined from all bins. The red straight line shows the estimated normalization offset $f = 2^{median(M_{A>q})}$. A 'smear' of yellow points at a low A value represents counts that are low in either of the two samples.

Figure S6 - Distribution of estimated methylation levels for SssI sample using Illumina HumanMethylation450 arrays

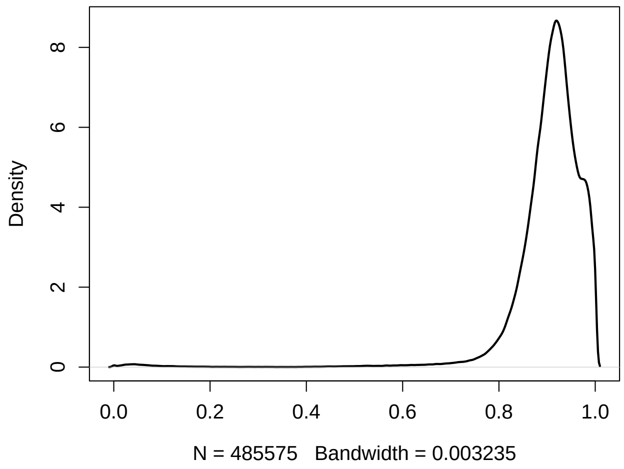

Density plot of 450k beta values.

Supplementary Table

Table S1 - Mean bias for IMR-90 analysis stratified by CpG island status and true methylation level

Mean bias for the 75% of bins with the largest depth in the truth (cutoff are 33 reads) for BayMeth, Batman, MEDIPS and BALM. Genomic regions are stratified by CpG-density using the threshold of 12.46 which separates CpG islands from non-CpG islands, compare Figure S1 of Additional file 2. Further stratification by the true methylation level as derived from WGBS is provided.

	No CpG-islands			CpG-islands		
Method	$[0, 0.2]$	$(0.2, 0.8]$	$(0.8, 1]$	$[0, 0.2]$	$(0.2, 0.8]$	$(0.8, 1]$
BayMeth	0.41	0.06	-0.22	0.09	0.14	-0.07
Batman	0.69	0.29	-0.01	0.11	0.15	-0.02
MEDIPS	0.05	-0.27	-0.54	0.05	-0.16	-0.47
BALM	-0.03	-0.37	-0.57	-0.01	-0.16	-0.31
