# Supplementary material for: BayMeth: improved DNA methylation quantification for affinity capture sequencing data using a flexible Bayesian approach
Source: Genome Biol. 2014 Feb 11;15(2):R35. doi: 10.1186/gb-2014-15-2-r35 (PMC4053803; doi:10.1186/gb-2014-15-2-r35)
Supplement: Additional file 3 — BayMeth analysis of Bock data. This document outlines all data preparation steps performed and presents detailed R code for the BayMeth analysis conducted using the Bioconductor package Repitools. [file gb-2014-15-2-r35-S3.pdf]

Additional file 3 — BayMeth analysis of “Bock” data

Andrea Riebler[1,2,3,*], Mirco Menigatti[4], Jenny Z. Song[5], Aaron L. Statham[5] , Clare Stirzaker[5,6], Nadiya Mahmud[7], Charles A. Mein[7], Susan J. Clark[5,6] , Mark D. Robinson[1,8,*]

[1]Institute of Molecular Life Sciences, University of Zurich, Winterthurerstrasse 190, CH-8057 Zurich, Switzerland
[2]Institute of Social- and Preventive Medicine, University of Zurich, Hirschengraben 84, CH-8001 Zurich, Switzerland
[3]Department of Mathematical Sciences, Norwegian University of Science and Technology, N-7491 Trondheim, Norway
[4]Institute of Molecular Cancer Research, University of Zurich, Winterthurerstrasse 190, CH-8057 Zurich, Switzerland
[5]Epigenetics Laboratory, Cancer Research Program, Garvan Institute of Medical Research, Sydney 2010, New South Wales, Australia
[6]St Vincent's Clinical School, University of NSW, Sydney 2052, NSW, Australia
[7]Genome Centre, Barts and the London, Queen Mary, University of London, Charterhouse Square, London EC1M 6BQ, United Kingdom
[8]SIB Swiss Institute of Bioinformatics, University of Zurich, Zurich, Switzerland

Email: Andrea Riebler*- andrea.riebler@math.ntnu.no; Mirco Menigatti - menigatti@imcr.uzh.ch; Jenny Z. Song - j.song@garvan.org.au; Aaron L. Statham- a.statham@garvan.org.au; Clare Stirzaker - c.stirzaker@garvan.org.au; Nadiya Mahmud - n.mahmud@qmul.ac.uk; Charles A. Mein - c.a.mein@qmul.ac.uk; Susan J. Clark - s.clark@garvan.org.au; Mark D. Robinson*- mark.robinson@imls.uzh.ch;

*Corresponding author

We applied (default) BayMeth to the MethylCap sequencing data of [1], provided at http://www.broadinstitute.org/labs/meissner/mirror/papers/meth-benchmark/index.html, and denoted as the “Bock” data below. Absolute read densities are available for four samples: HUES6 ES cell line, HUES8 ES cell line, colon tumor tissue, colon normal tissue (same donor as for colon tumor tissue), based on hg18 and given for (non-overlapping) 50bp bins. There is no matched SssI sample available for these data. To take advantage of BayMeth in analyzing these data, we use a non-matching SssI sample, but one chosen to be maximally compatible to the preparation conditions of Bock data [1] (i.e. MethylCap at low salt concentration: 200mM NaCl). Furthermore, RRBS data are available for each sample representing absolute DNA methylation levels at single CpGs.

In section 1, we outline all data preparation steps. First, all samples of interest are saved in a single GRanges object based on genome-wide non-overlapping 50bp bins. RRBS information is loaded and saved in the same object. Since the read density for the fully methylated sample is based on hg19, the Bock data are lifted over. Based on hg19 we derive CpG density and mappability estimates. Finally, all information is stored in a BayMethList data object. Section 2 describes the BayMeth analysis applied on the former created BayMethList data object. Normalizing offsets are derived for all samples, before the empirical Bayes approach is used to get suitable prior parameters. Finally region-specific methylation estimates are

	HUES6	HUES8	Colon_normal	Colon_tumor
Min.	0.00	0.00	0.00	0.00
1st Qu.	0.00	0.00	0.00	0.00
Median	0.00	0.00	0.00	0.00
Mean	2.00	1.81	1.96	1.99
3rd Qu.	2.00	2.00	2.00	2.00
Max.	374.00	400.00	407.00	400.00

Table 1: Summary information for absolute read counts for each sample.

computed.

1 Data preparation

1.1 Samples of interest

We applied BayMeth to the MethylCap sequencing data of [2]. Data are available for four samples: 1) HUES6 ES cell line, 2) HUES8 ES cell line, 3) Colon tumor tissue, 4) Colon normal tissue (same donor as (3)). Absolute read densities provided as bigwig files were downloaded, converted to `GRanges` objects and saved in a `GRangesList`:

```
setwd("./4_bock/")
library(rtracklayer)
data_names <- c("HUES6", "HUES8", "Colon_normal", "Colon_tumor")
grl_bock_methylCap <- GRangesList()
for(i in 1:length(data_names)){
    print(data_names[i])
    # import the data and convert to GRanges
    data_tmp <- import(paste("data/ChIP_absReadFreqW50_MethylCap_", data_names[i], "_all.bw", sep=""),"bw")
    data_tmp <- as(data_tmp,"GRanges")
    grl_bock_methylCap <- c(grl_bock_methylCap, GRangesList(data_tmp))
}
```

Read densities are based on (non-overlapping) 50bp bins. Summary information for each sample is shown in Table 1.

```
sumTab <- cbind(summary(values(grl_bock_methylCap[[1]])$score),
summary(values(grl_bock_methylCap[[2]])$score),
summary(values(grl_bock_methylCap[[3]])$score),
summary(values(grl_bock_methylCap[[4]])$score))
```

Of note, read density information for the different samples is not given for the same bins. To save all data in one `GRanges` object, a genome-wide GRanges object for hg18 based on non-overlapping 50bp was created.

```
library(BSgenome.Hsapiens.UCSC.hg18)
library(Repitools)
library(GenomicRanges)
# save all datasets in one GRanges object
gb_hg18 <- genomeBlocks(Hsapiens, 1:24, width=50)
#
tumor <- normal <- hues6 <- hues8 <- rep(NA, length(gb_hg18))
#
fo_hues6 <- findOverlaps(gb_hg18, grl_bock_methylCap[[1]])
fo_hues8 <- findOverlaps(gb_hg18, grl_bock_methylCap[[2]])
fo_normal <- findOverlaps(gb_hg18, grl_bock_methylCap[[3]])
fo_tumor <- findOverlaps(gb_hg18, grl_bock_methylCap[[4]])
#
inds_hues6 <- split(fo_hues6@subjectHits, fo_hues6@queryHits)
ind_hues6 <- as.integer(names(inds_hues6))
hues6[ind_hues6] <- values(grl_bock_methylCap[[1]])$score[fo_hues6@subjectHits]
#
inds_hues8 <- split(fo_hues8@subjectHits, fo_hues8@queryHits)
ind_hues8 <- as.integer(names(inds_hues8))
hues8[ind_hues8] <- values(grl_bock_methylCap[[2]])$score[fo_hues8@subjectHits]
#
# ... analogously for normal and tumor

df <- DataFrame("hues6"=hues6, "hues8"=hues8, "normal"=normal, "tumor"=tumor)
values(gb_hg18) <- df
```

To do this properly we have to ensure that the bins of [2] start at $1, 51, 101, 151, \ldots$ and have a width of 50bp. We have proved this using a modulo operation `table(start(grl_bock_methylCap[[i]]) %% 50)` which resulted in 1 for all bins, and `table(width(grl_bock_methylCap[[i]]))`, which resulted in 50 for all bins. Using the function `findOverlaps` the different read counts are saved as metadata at the corresponding positions in the object `gb_hg18`. If no information is provided for a bin, the read density is set to `NA`.

1.2 Reduced representation bisulphite sequencing (RRBS) information

Information on RRBS data are available on
http://www.broadinstitute.org/labs/meissner/mirror/papers/meth-benchmark/RRBS/, and used as gold standard in the following analysis. In the RRBS data for HUES6 and HUES8 we removed lines where the strand information is neither ”+” , ”-” nor ”*”, but ”b”, and saved the data in
RRBS_cpgMethylation_HUES6_strandCleaned.RRBS.bed and
RRBS_cpgMethylation_HUES8_strandCleaned.RRBS.bed, respectively.

Both, the number of reads that overlay a cytosine (T) and the number of cytosines that stay a cytosine (M), i.e. are methylated, are given. Note, that for one CpG site there is only information from one strand available.

```
data_names <- c("HUES6_strandCleaned", "HUES8_strandCleaned", "Colon_normal", "Colon_tumor")
# create container to save datasets
grl_bock_rrbs <- GRangesList()
for(i in 1:length(data_names)){
    # import the data and convert to GRanges
    data_tmp <- import(paste("data/RRBS_cpgMethylation_", data_names[i], ".RRBS.bed", sep=""),"BED")
    data_tmp <- as(data_tmp,"GRanges")

    # extract the number of reads that overlay a cytosine and the number
    # of cytosines that stay a cytosine i.e. are methylated
    name <- values(data_tmp)$name
    cpg <- strsplit(name, "/")
    cpg <- do.call(rbind, cpg)
    cpg <- sapply(1:ncol(cpg), function(u){as.numeric(cpg[,u])})
    colnames(cpg) <- c("numMeth", "total")

    # add the corresponding columns to the GRanges object
    # (meth correponds approximately to score/1000)
    values(data_tmp) <- cbind(values(data_tmp),
                            DataFrame(cpg, meth=cpg[,1]/cpg[,2]))

    grl_bock_rrbs <- c(grl_bock_rrbs, GRangesList(data_tmp))
}
names(grl_bock_rrbs) <- c("HUES6", "HUES8", "Colon_normal", "Colon_tumor")
```

To get smooth methylation estimates, we summarized CpG based RRBS data within 150bp bins (overlapping by 100bp). The methylation level for one 150bp bin i is thereby derived as:

$$m_i = \frac{\sum M_{\in i}}{\sum T_{\in i}}.$$

That means using information for all CpG sites that fall into bin i.

```
gb_hg18_150 <- resize(gb_hg18, 150, fix="center")
# get the corresponding rrbs estimates
meth_names <- c("rrbs_hues6_meth", "rrbs_hues8_meth", "rrbs_normal_meth", "rrbs_tumor_meth")
denom_names <- c("rrbs_hues6_denom", "rrbs_hues8_denom","rrbs_normal_denom", "rrbs_tumor_denom")
for(i in 1:4){
    rrbs_tmp <- grl_bock_rrbs[[i]]
    fo_tmp <- findOverlaps(gb_hg18_150, rrbs_tmp)
    inds_tmp <- split(fo_tmp@subjectHits, fo_tmp@queryHits)

    nmeth <- values(rrbs_tmp)$numMeth
    total <- values(rrbs_tmp)$total

    methI <- sapply(inds_tmp, function(u) sum(nmeth[u])/sum(total[u]))
    denomI <- sapply(inds_tmp, function(u) sum(total[u]))

    denom <- meth <- rep(NA, length(gb_hg18))
    # assign the derived estimates to the corresponding genomic bins
    ind_tmp <- as.integer(names(inds_tmp))
    meth[ind_tmp] <- methI
    denom[ind_tmp] <- denomI
    tmp_df <- DataFrame(meth, denom)
    colnames(tmp_df) <- c(meth_names[i], denom_names[i])
    values(gb_hg18) <- cbind(values(gb_hg18), tmp_df)
}
```

Figure 1 shows a smooth density representation of the RRBS methylation estimates versus the MethylCap read density after filtering bins where no truth exists and only taking a minimum depth of 20 in RRBS.

1.3 Lift-over to hg19

Since the data for the fully methylated (SssI treated) sample are based on hg19, the bin coordinates of hg18 are transferred to the corresponding position on hg19.

```
chain <- import.chain("data/hg18ToHg19.over.chain")
gb_hg19 <- liftOver(gb_hg18, chain)
gb_hg19 <- unlist(gb_hg19)
```

We remove all bins with a width unequal to 50bp.

```
library(BSgenome.Hsapiens.UCSC.hg19)
w.idx <- which(width(gb_hg19) != 50)
gb_hg19r <- gb_hg19[-w.idx]
```

Lifting the bins over to hg19 caused overlapping bins. Hence, we remove all bins that have more than one overlap (namely with itself).

```
fo <- findOverlaps(gb_hg19r, gb_hg19r)
inds <- split(fo@subjectHits, fo@queryHits)
len <- unlist(lapply(inds, length))
w2.idx <- which(len != 1)
gb_hg19r <- gb_hg19r[-w2.idx]
```

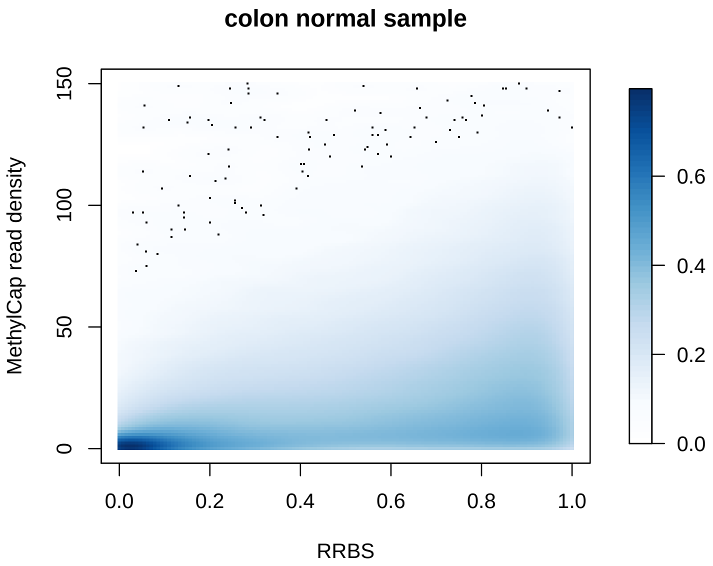

Figure 1: Comparison between read frequencies and DNA methylation levels derived from RRBS for the colon normal sample. Unprocessed read frequencies for MethylCap were correlated with DNA methylation levels as determined by RRBS.

1.4 SssI sample, CpG density and mappability information

BayMeth quantifies methylation of an affinity-enrichment sequencing dataset best by taking advantage of a full methylated control data set. Here, we use a sample treated with SssI and analysed using MethylCap at low salt concentration, i.e., 200 mM NaCl, to be maximally compatible to the preparation conditions of [1].

```
library(BSgenome.Hsapiens.UCSC.hg19)
f <- "data/SSSl_low.bam"
names(f) <- "SssI_low"
counts <- annotationBlocksCounts(f, gb_hg19r, seq.len=150)
```

The CpG density is calculated by symmetrically extending the bins around the bin center to a length of 700bp and linear weighting the CpG sites falling into this range.

```
gbA <- resize(gb_hg19r, 1, fix="center")
cpgdens <- cpgDensityCalc(gbA, organism=Hsapiens, w.function="linear", window=700)
```

Mappability probabilities are derived from http://hgdownload.cse.ucsc.edu/goldenPath/hg19/encodeDCC/wgEncodeMapability/wgEncodeCrgMapabilityAlign50mer.bigWig.

```
library(rtracklayer)
bw <- BigWigFile("data/wgEncodeCrgMapabilityAlign50mer_hg19.bigWig")
map <- import(bw)
score <- score(map)
wd <- width(map)
fo <- findOverlaps(gb_hg19r, map)
ind <- split(fo@subjectHits,fo@queryHits)
mapv <- numeric(length(gb_hg19r)) # default of 0
w <- as.numeric(names(ind))
# take weighted mean
mapv[w] <- sapply(ind, function(u) sum( wd[u]*score[u] ) / sum(wd[u]) )
values(gb_hg19r) <- cbind(values(gb_hg19r), DataFrame("cpgdens"=cpgdens, "map_ucsc"=mapv, "SssI-low"=counts))
save(gb_hg19r, file="data/bock_data_prepared.Rdata")
```

SssI read densities, CpG density and mappability are saved as further metadata columns in gb_h19r.

2 BayMeth Analysis

Here, my session info:

```
sessionInfo()
#R Under development (unstable) (2013-07-03 r63169)
#Platform: x86_64-unknown-linux-gnu (64-bit)
#
#locale:
# [1] LC_CTYPE=en_CA.UTF-8       LC_NUMERIC=C
# [3] LC_TIME=en_US.UTF-8        LC_COLLATE=en_CA.UTF-8
# [5] LC_MONETARY=en_US.UTF-8    LC_MESSAGES=en_CA.UTF-8
# [7] LC_PAPER=en_US.UTF-8       LC_NAME=C
# [9] LC_ADDRESS=C               LC_TELEPHONE=C
#[11] LC_MEASUREMENT=en_US.UTF-8 LC_IDENTIFICATION=C
#
#attached base packages:
#[1] parallel  stats     graphics  grDevices utils     datasets  methods
#[8] base
#
#other attached packages:
# [1] lattice_0.20-15                   fields_6.7
# [3] spam_0.29-3                       Repitools_1.7.13
# [5] BSgenome.Hsapiens.UCSC.hg18_1.3.19 BSgenome_1.29.1
# [7] Biostrings_2.29.15                rtracklayer_1.21.9
# [9] GenomicRanges_1.13.36             XVector_0.1.0
#[11] IRanges_1.19.24                   BiocGenerics_0.7.4
#
#loaded via a namespace (and not attached):
# [1] bitops_1.0-6       edgeR_3.3.7        grid_3.1.0         KernSmooth_2.23-10
# [5] limma_3.17.21      RCurl_1.95-4.1     Rsamtools_1.13.29  Rsolnp_1.14
# [9] stats4_3.1.0       tools_3.1.0        truncnorm_1.0-6    XML_3.98-1.1
#[13] zlibbioc_1.7.0
```

We start the analysis by loading the data. We remove bins with zero reads in all four samples and in the control, and generate a `BayMethList` object. This object is initialized with four entries:

- `windows`: A `GRanges` object representing the genomic bins of interest.
- `control`: A `matrix` of read counts obtained by an affinity enrichment sequencing experiment for the fully methylated (SssI) treated sample. The number of rows must be equal to 'length(windows)'. Each column contains the counts of one sample. The number of columns must be either one or equal to the number of columns of 'sampleInterest'.
- `sampleInterest`: A matrix of read counts obtained by an affinity enrichment sequencing experiment for the samples of interest. The number of rows must be equal to 'length(windows)'. Each column contains the counts of one sample.
- `cpgDens`: A numeric vector containing the CpG density for 'windows'. The length must be equal to

'length(windows)'

```
library(Repitools)
# load the prepared data object
load("data/bock_data_prepared.Rdata")
metDat <- as.matrix(values(gb_hg19r))
# remove bins where we have no read depth in none of the samples
rs <- rowSums(metDat[, c("hues6", "hues8", "normal", "tumor", "SssI.low.SssI_low")])
wr <-  which(rs == 0)
gb_hg19_noZero <- gb_hg19r[-wr]
metDat <- metDat[-wr,]
map <- metDat[, "map_ucsc"]
sssI <- matrix(metDat[,"SssI.low.SssI_low"], ncol=1)
colnames(sssI) <- "sssI"
bockBL <- BayMethList(
    window=window(gb_hg19_noZero),
    control=sssI,
    sampleInterest=cbind(hues6=metDat[,"hues6"], hues8=metDat[,"hues8"],
                    normal=metDat[,"normal"], tumor=metDat[,"tumor"]),
    cpgDens=metDat[,"cpgdens"])
```

We only include autosomes in the analysis and concentrate on bins with with at least 75% mappable bases.

```
# only consider autosomes
as.idx <- !(seqnames(windows(bockBL)) %in% c("chrX", "chrY"))
as.idx <- as.vector(as.idx)
bockBL <- bockBL[as.idx]
map <- map[as.idx]
bockBL <- bockBL[map > 0.75]
```

Next, we determine the normalizing constant for each sample. The normalizing factor f is essentially a scaling factor between highly methylated regions in the corresponding sample relative to the SssI control, see Figure 2.

```
bockBL <- determineOffset(bockBL, q=0.998, controlPlot=list(show=TRUE, mfrow=c(2,2), nsamp=100000,
   main=colnames(sampleInterest(bockBL)), ask=FALSE))
fOffset(bockBL)
#        hues6 hues8   normal    tumor
#[1,] 2.289898  2.75 1.285714 1.272727
```

Using the empirical Bayes approach we have to be aware of bins with unusual high counts of reads. These might cause problems in the optimization routine as they can cause `NA` or `Inf` values returned by the hypergeometric function. Some of these high read counts can be explained by unannotated high copy number regions, see [3]. We mask these bins out for the empirical Bayes procedure to avoid numerical problems. However, note that we will finally obtain methylation estimates for almost all of these bins.

```
## mask suspicious regions
#wget http://eqtl.uchicago.edu/Masking/seq.cov1.ONHG19.bed.gz
library(rtracklayer)
hcRegions <- import("data/seq.cov1.ONHG19.bed", asRangedData=FALSE)
bockBL <- maskOut(bockBL, hcRegions)
```

Using this reduced dataset we derive the prior parameters based on empirical Bayes. We use a uniform

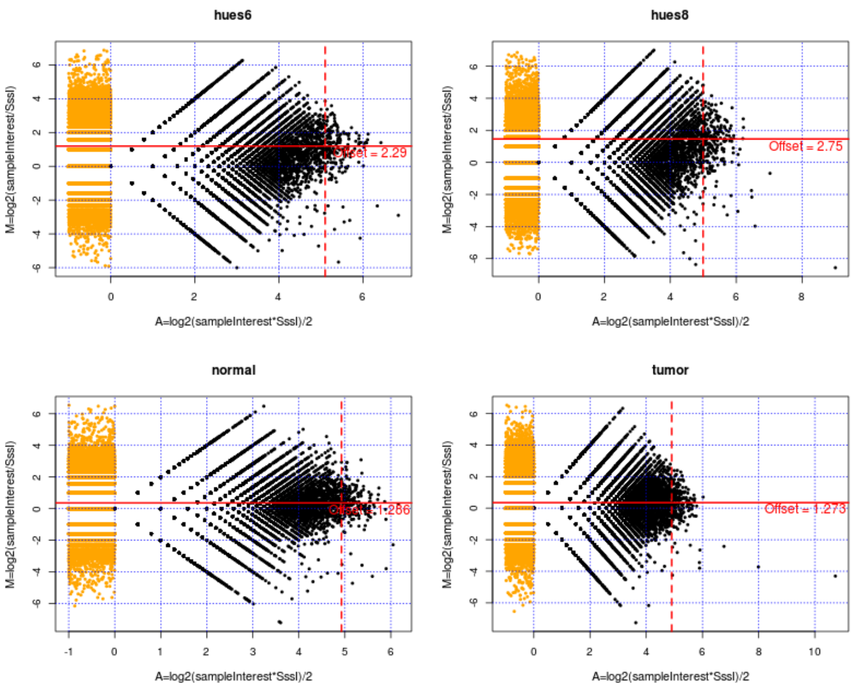

Figure 2: Log-fold change (M) versus log-concentration (A) illustrated for all four samples randomly sampling data of 100000 bins in each case. The red dotted line shows the 0.998 quantile q of A determined from all bins. The red straight line shows the estimated normalization offset $f = 2^{median(M_{A>q})}$. A 'smear' of yellow points at a low A value represents counts that are low in either of the two samples.

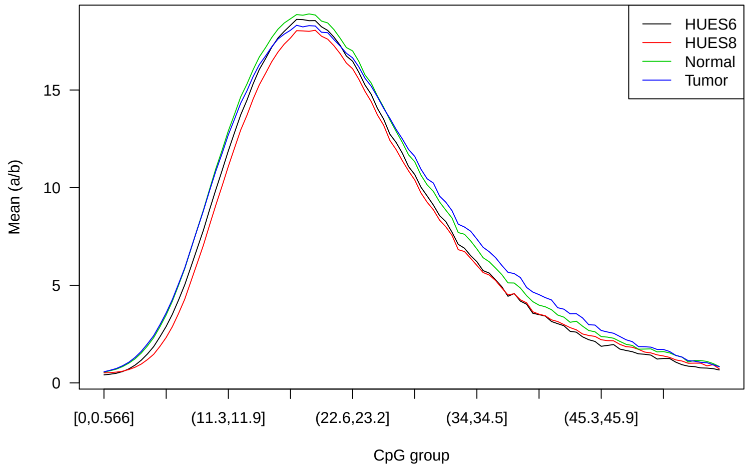

Figure 3: Mean of the prior predictive distribution depending on CpG density group for all four samples.

prior distribution for the methylation level and consider $K = 100$ separate CpG groups. The algorithm is run on four CPUs in parallel.

```
## find prior parameters using empirical Bayes
bockBL <- empBayes(bockBL, ngroups = 100, ncomp = 1, maxBins = 50000,
  method="beta", ncpu=4, verbose=FALSE)
```

The prior parameters for all samples are saved in a list, which can be accessed using the function `priorTab(.)`. The first list element contains a vector with the assigned CpG density group for each bin. Of note, the length of this vector is equal to the numbers of bins used in the analysis. The second list element saves the number of mixture components used and the third contains a string indicating the type of prior (”beta” or ”DBD”). The following entries contain the prior parameters for each sample. One list element corresponds thereby to one sample. Figure 3 shows the mean of the obtained prior predictive distribution of the SssI sample depending on CpG density group for all four samples.

```
plot(priorTab(bockBL)[[4]][1,]/priorTab(bockBL)[[4]][2,],
  type="l", xlab="CpG group", ylab="Mean (a/b)", xaxt="n")
axis(1, at=seq(1,100,10), labels=levels(priorTab(bockBL)[[1]])[seq(1,100,10)])
for(i in 2:4){
    lines(priorTab(bockBL)[[3+i]][1,]/priorTab(bockBL)[2,], type="l", col=i)
}
legend("topright", c("HUES6", "HUES8", "Normal", "Tumor"), lty=1, col=1:4)
```

To get methylation estimates we call:

```
bockBL <- methylEst(bockBL, verbose=TRUE, controlCI = list(compute = FALSE))
```

This function assigns a list to the slot `methEst` in our `BayMethList` object. Here, the mean, variance and potential credible intervals are saved for each sample. The mean and variance can be accessed using

`methEst(bockBL)$mean` and `methEst(bockBL)$var` .

Figure 4 shows regional methylation estimates of BayMeth compared to RRBS for all samples. Note this figure is the same as Figure 9 of the main text.

```
mE <- methEst(bockBL)$mean
mV <- methEst(bockBL)$var
cP <- cpgDens(bockBL)
sssI <- control(bockBL)
sI <- sampleInterest(bockBL)
## get the truth for all samples
rrBS <- as.matrix(values(windows(bockBL))[,5:12])
rrBS <- as.matrix(rrBS)
#
# combine everything in one matrix to facilitate plotting
all <- cbind(mE, rrBS, cP, sssI, sI, mV)
colnames(all) <- c("bayMeth_hues6", "bayMeth_hues8", "bayMeth_normal", "bayMeth_tumor",
  colnames(rrBS), "cpgDens", "sssI", "hues6", "hues8", "normal", "tumor",
  "bayMeth_varHues6", "bayMeth_varHues8", "bayMeth_varNormal", "bayMeth_varTumor")
#
sNames <- c("a) HUES6", "b) HUES8", "c) Colon normal", "d) Colon tumor")
#
alls <- all
#
col <- "dodgerblue4"
Lab.palette <- colorRampPalette(c("blue", "orange", "red"), space = "Lab")
par(mfrow=c(2,2), mar=c(3.5,4, 3, 4.5), mgp=c(2.5,1,0), cex.lab=.85, cex.main=1, cex.axis=.75, pty="s", las=1)
zlim <- c(0,2.34)
lim <- c(0,1)
for(i in 1:4){
  all <- alls
  all <- all[!is.na(all[,5+2*(i-1)]),]
  all <- all[!is.na(all[,i]),]
  #
  ## define a limit for the truth
  limit_truth <- 20
  all <- all[all[,6+2*(i-1)] > limit_truth,]
  #
  ## separation by variance
  limit_var <- 0.0225
  all <- all[all[,19+(i-1)] < limit_var,]
  #
  ## separation by SssI control
  limit_control <- 9
  all <- all[all[,"sssI"] > limit_control,]
  #
  ## smooth density representation
  mysmoothScatter(all[,5+2*(i-1)], all[,i], pch=".",
    col=col, colramp=Lab.palette, xlab="RRBS", ylab="BayMeth",
    main=sNames[i], xlim=lim, ylim=lim,
    cex=0.05, horizontal=F, zlim=zlim,
    axis.args=list(at=zlim, labels=c("low", "high")))
  text(0.5, 0.05, sum(!is.na(all[,i])), col="white", cex=0.85)
  abline(c(0,0), c(1,1), col="green", lwd=1.3, lty=2)
}
```

Here, `mysmoothScatter` represents an adaptation of the function `smoothScatter` to get a color key next to the figures.

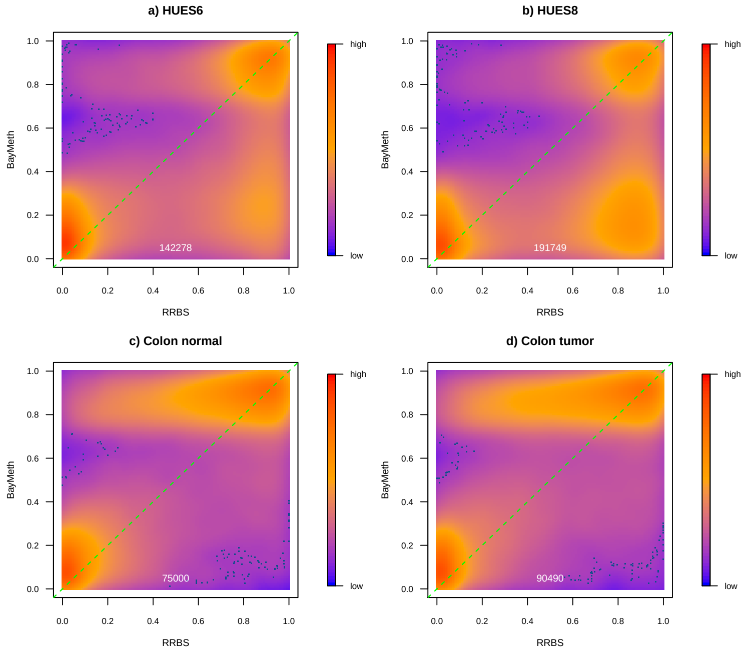

Figure 4: Smooth color density representation of variance estimates obtained by BayMeth versus number of reads in the SssI control for a read depth larger than 20 in RRBS. The red box contains the bins used in Figure 9 having at least a depth of 10 in SssI and a standard deviation smaller than 0.15, i.e. a variance smaller than 0.025.

References

1. Bock C, Tomazou EM, Brinkman A, Müller F, Simmer F, Gu H, Jäger N, Gnirke A, Stunnenberg HG, Meissner A: **Genome-wide mapping of DNA methylation: a quantitative technology comparison**. *Nature Biotechnology* 2010, **28**:1106–1114.
2. Bock C, Tomazou E, Brinkman A, Müller F, Simmer F, Gu H, Jäger N, Gnirke A, Stunnenberg H, Meissner A: **Quantitative comparison of genome-wide DNA methylation mapping technologies**. *Nature Biotechnology* 2010, **28**(10):1106–1114.
3. Pickrell J, Gaffney D, Gilad Y, Pritchard J: **False positive peaks in ChIP-seq and other sequencing-based functional assays caused by unannotated high copy number regions**. *Bioinformatics* 2011, **27**(15):2144–2146.
